# Pulsed‐Current Operation Enhances H_2_O_2_ Production on a Boron‐Doped Diamond Mesh Anode in a Zero‐Gap PEM Electrolyzer

**DOI:** 10.1002/cssc.202401947

**Published:** 2025-01-16

**Authors:** Adam Vass, Maximilian Göltz, Hanadi Ghanem, Stefan Rosiwal, Tanja Franken, Regina Palkovits, Guido Mul, Mihalis N. Tsampas, Georgios Katsoukis, Marco Altomare

**Affiliations:** ^1^ Department of Chemical Engineering MESA+ Institute for Nanotechnology Faculty of Science and Technology University of Twente Drienerlolaan 5 7522 NB Enschede, The Netherlands; ^2^ Friedrich-Alexander-Universität Erlangen-Nürnberg Schloßplatz 4 91054 Erlangen Germany; ^3^ Forschungszentrum Jülich Institute for a Sustainable Hydrogen Economy (INW-2) Marie-Curie- Straße 5 52428 Jülich Germany; ^4^ Institute for Technical and Macromolecular Chemistry RWTH Aachen University Worringerweg 2 52074 Aachen Germany; ^5^ Max-Planck-Institute for Chemical Energy Conversion Stiftstraße 34–36 45470 Mülheim an der Ruhr Germany; ^6^ Dutch Institute for Fundamental Energy Research (DIFFER) 5612AJ Eindhoven, The Netherlands

**Keywords:** hydrogen peroxide, boron-doped diamond, zero-gap, PEM, pulsed current electrolysis

## Abstract

A niobium (Nb) mesh electrode was coated with boron‐doped diamond (BDD) using chemical vapor deposition in a custom‐built hot‐filament reactor. The BDD‐functionalized mesh was tested in a zero‐gap electrolysis configuration and evaluated for the anodic formation of H_2_O_2_ by selective oxidation of water, including the analysis of the effects on Faradaic efficiency towards H_2_O_2_ $\left({\mathrm{FE}}_{H_{2}O_{2}}\right)$ induced by pulsed electrolysis. A low electrolyte flow rate (${\dot{V}}_{\mathrm{anolyte}}$ ) was found to result in a relatively high concentration of H_2_O_2_ in single‐pass electrolysis experiments. Regarding pulsed electrolysis, we show an optimal ratio of on‐time to off‐time to obtain the highest concentration of H_2_O_2_. Off‐times that are “too short” result in decreased ${\mathrm{FE}}_{H_{2}O_{2}}$ , whereas “too long” off‐times dilute the product in the electrolyte stream. Using our electrolyzer setup with an anodic pulse of 2 s with 4 s intervals, and a ${\dot{V}}_{\mathrm{anolyte}}$ of 0.75 cm^3^ min^−1^, resulted in the best performance. This adjustment increased the ${\mathrm{FE}}_{H_{2}O_{2}}$ by 70 % compared to constant current electrolysis, at industrially relevant current densities (150 mA cm^−2^). Fine tuning of BDD morphology, flow patterns, and anolyte composition might further increase the performance of zero‐gap electrolyzers in pulsed operation modes.

## Graphical abstract

Pulsed current electrolysis with a boron‐doped diamond coated mesh anode in a zero‐gap electrolyzer increases the Faradaic efficiency of H_2_O_2_ formation by 70 % compared to constant current electrolysis at industrially relevant current density.

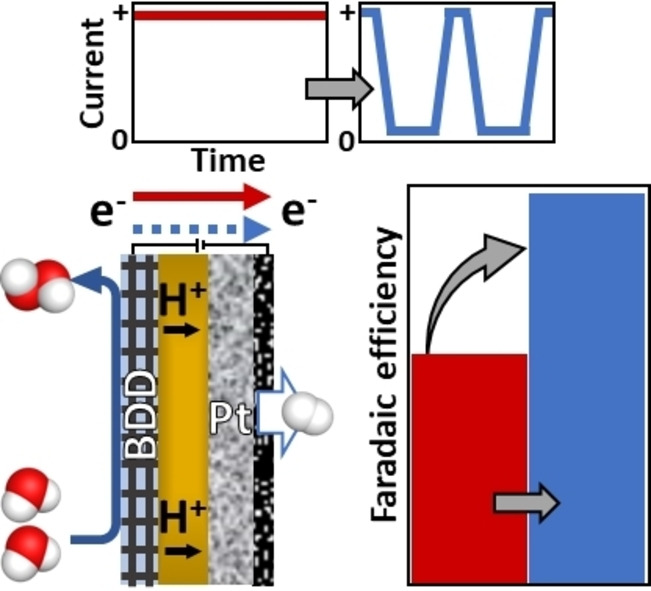

## 1 Introduction

Hydrogen peroxide, H_2_O_2,_ is a green oxidant which is widely used in several industrial applications such as bleaching, water treatment, sanitization and chemical synthesis, and has a growing market value.^[1]^ H_2_O_2_ is mainly produced industrially through the anthraquinone autoxidation process, which is energy‐demanding, needs large amounts of organic solvents, and carries remarkable safety risks due to hydrogenation and oxidation reactions at high pressure. In addition, the anthraquinone process requires expensive metal catalysts and involves distillation steps to generate large volumes of concentrated solutions. The latter steps are needed for the subsequent transportation of H_2_O_2_ to points of use, where H_2_O_2_ is diluted to the desired concentration.^[2]^

In this framework, the electrochemical synthesis of H_2_O_2_ using renewable electricity and naturally abundant feedstock (H_2_O, O_2_) is a sustainable alternative to the anthraquinone process. Moreover, the electrochemical synthesis of H_2_O_2_ can potentially be implemented in a decentralized way, where H_2_O_2_ is produced directly at points of use with the required concentration.[^3^, ^4^] There are two possible electrochemical pathways for H_2_O_2_ production: i) the cathodic O_2_ reduction reaction (ORR);^[5]^ and ii) the anodic H_2_O oxidation reaction (WOR) – both pathways proceed via a two‐electron transfer reaction.^[6]^ The latter, i. e., WOR, is attractive particularly because it can be coupled with valuable cathodic reactions such as the H_2_ evolution reaction (HER), CO_2_ reduction reaction, or the production of H_2_O_2_ via ORR – the latter enables both anodic and cathodic half‐cell reactions to synthesize the same target product, i. e., H_2_O_2_.[^7^, ^8^, ^9^]

Most studies on the anodic production of H_2_O_2_ have tested various catalysts and electrode materials, in batch reactors, and using primarily H‐cell setups.[^10^, ^11^, ^12^, ^13^, ^14^] The challenge in batch electrolysis is to prevent further oxidation of the produced H_2_O_2_ to O_2_. In fact, in a batch reactor process, the formed H_2_O_2_ accumulates until its concentration increases to a critical value around which the rate of oxidation of H_2_O_2_ (to O_2_) counterbalances the H_2_O_2_ formation rate. This limits the H_2_O_2_ concentration to increase further and causes losses in Faradaic efficiency (${\mathrm{FE}}_{H_{2}O_{2}}$ ). Flow‐type electrolyzers have gained attention recently because, in a flow cell, fresh electrolyte is continuously fed to the electrode, e. g., in single‐pass operation. This minimizes the product′s residence time and thus avoids the (electro)chemical oxidation of the product (H_2_O_2_).[^7^, ^8^, ^15^, ^16^] Flow‐cell configurations reported to date feature a distance between the electrodes and the separator (e. g., ion exchange membrane), where the liquid electrolyte flows by the electrode surface. In such cells, typical electrode materials for WOR towards H_2_O_2_ formation are fluorine‐doped tin oxide or boron‐doped diamond (BDD), usually in the form of thin films on solid (non‐porous) electrode substrates. Among other electrode materials, boron‐doped diamond is promising for H_2_O_2_ formation via water oxidation due to various reasons, i. e.: its robustness and stability in a wide range of electrochemical conditions, including alkaline or acidic pH, and at high anodic or cathodic potentials; it is composed of carbon, hence of earth abundant materials; it has a high selectivity towards H_2_O_2_ formation, and high overpotential for OER. Research efforts on BDD for the anodic production of H_2_O_2_ have mainly focused on optimizing the physicochemical properties of the material, mostly using BDD‐coated planar electrodes. Little effort, however, has been made in the direction of cell design development.^[17]^ Only for water treatment, BDD has been utilized in diverse cell configurations.^[18]^ For instance, in the filter‐press‐type FM01‐LC electrochemical reactor, 3D BDD meshes have been tested for mineralization of organic compounds.^[19]^

A zero‐gap membrane electrode assembly (MEA) design based on porous electrodes is also desirable for anodic production of H_2_O_2_ to minimize the ohmic resistances and lower the cell voltage, hence, to increase the process energy efficiency. In the present study we investigate a cell design based on a zero‐gap PEM electrolyzer with a BDD‐coated metal mesh anode.^[20]^ We demonstrate the feasibility of anodic H_2_O_2_ formation by partial WOR in a flow‐cell configuration, pairing the anode reaction with HER on the cathode side. In addition, we show that pulsed electrolysis significantly enhances the H_2_O_2_ formation efficiency. Particularly, we systematically investigated the on‐time to off‐time ratio and the amplitude of the current‐pulse cycles and achieved a 70 % increase in Faradaic efficiency to H_2_O_2_ compared to constant‐current electrolysis, at industrially relevant current densities (i. e.,150 mA cm^−2^). The choice of a BDD anode is, as introduced earlier, based on several studies demonstrating that BDD has excellent catalytic properties for formation of H_2_O_2_.[^13^, ^14^, ^21^, ^22^, ^23^, ^24^, ^25^]

## 2 Experimental

### 2.1 Chemicals and Materials

All chemicals and reagents utilized in this research were analytical grade and were used without further purification. H_2_O (Milli‐Q, 18.2 MΩ at 25 °C), K_2_CO_3_ (≥99.0 %, Sigma‐Aldrich), H_2_O_2_ (30 wt.%, Sigma‐Aldrich), H_2_SO_4_ (95.0–98.0 %, Sigma‐Aldrich), TiOSO_4_ (15 wt.% in dilute H_2_SO_4_, 99.99 % trace metals basis, Sigma‐Aldrich). Sigracet 22 BB carbon paper (Fuel Cell Store), Pt target (99.99 %, AJA International Inc., USA), Nafion 117 purchased from Ion‐Power GmbH (Dupont), He (5.0, Hinnen).

### 2.2 Preparation and Characterization of BDD Mesh Anode

The H_2_O_2_ formation electrode used in this study was a boron‐doped diamond (BDD) layer coated on an expanded Nb metal mesh substrate (Metakem). The BDD coating was deposited in a custom‐built hot‐filament chemical vapor deposition reactor (HF‐CVD) with a capacity of 1 m^2^, where the substrate was placed between two rows of tungsten filaments.^[14]^ The atmosphere consisted of hydrogen with 4 V/V % methane and 0.003 V/V % trimethyl borate as a dopant precursor, the pressure was 3 mbar; the substrate temperature was about 800 °C. After the deposition, not further modification of the BDD/Nb electrode was carried out.

Suitable substrates for doped diamond film deposition are usually either silicon or self‐passivating metals such as titanium, tantalum, tungsten, molybdenum and niobium. Niobium is best suited because it can withstand the deposition conditions (high temperature in a hydrogen containing atmosphere) with the least changes in the material properties. For many practical applications, including the fabrication of electrodes shown in our study, the brittleness of silicon is problematic. Hence, we opted for doped‐diamond films on Nb metal substrates. Other advantages of Nb metal substrates are the possibility to produce porous and scalable electrodes, with size up to the square meter range, together with the electrochemical stability of Nb under anodic conditions.

Scanning electron microscopy (SEM) imaging was performed using a FEI Quanta 450 scanning electron microscope. Glow discharge optical emission spectroscopy (GDOES) analysis was used to determine the concentration of boron doping using a HORIBA Scientific GD‐Profiler 1 calibrated by mass spectrometry standards. The Raman spectrum of BDD was recorded using a WITec alpha300 R confocal microscope with a 532 nm, 15 mW laser and a grating of 1800 grooves mm^−1^.

### 2.3 Preparation of Cathode GDE

Pt films were deposited on carbon paper gas diffusion layers (GDL, Sigracet 22 BB) to obtain cathode gas diffusion electrodes (GDEs) using a magnetron sputter coater (ATC Polaris, AJA International Inc., USA), equipped with a Pt target (99.99 % purity, AJA International Inc., USA) and powered by an RF power supply (Power source 0313GTC, T&C Power, USA). The working pressure during sputtering was maintained at 4.2×10^−6^ bar.^[26]^

Pt films of 50 nm (nominal thicknesses) were obtained with a sputtering time of 11 min while keeping the plasma power at 50 W, yielding a constant deposition rate of 4.5 nm min^−1^.

The sputtering rate was previously determined by depositing Pt films (sputtering power of 50 W) of different thicknesses, i. e., different sputtering times, on Si wafers. The thickness of these films was determined by X‐ray reflectivity (XRR).

### 2.4 Electrolysis

The H_2_O_2_ formation electrolysis experiments were performed in a custom made zero‐gap electrolyzer made of titanium endplates with serpentine flow channels. The anodic H_2_O_2_ formation was paired with hydrogen evolution reaction (HER) on the cathode side.

The membrane electrode assembly (MEA) consisted of a boron‐doped diamond (BDD) coated Nb mesh as anode electrode; 50 nm Pt sputter‐deposited on carbon paper GDL for the cathode GDE; and a Nafion 117 cation exchange membrane as separator (Figure S1A). The size of the electrodes was 38 mm×38 mm (Figure S1B). We used ImageJ software to estimate, approximately, the area of the contact between the surface of the mesh anode and the membrane, and the surface area of the sides/walls of the holes, see Figure S1C and D respectively. The values are approximately 1 cm^2^ for the contact surface and 0.57 cm^2^ for the sides of the holes (Σ_perimeter_×height). However, as it is difficult to calculate the surface area of such mesh electrode precisely, we used the nominal geometric area of the electrodes (14.44 cm^2^) to estimate the geometric current density values.

The anodic electrolyte (anolyte) was 2 M K_2_CO_3_ solution (pH=12.36) for the following reasons: (i) the positive influence of carbonate ions (${\mathrm{CO}}_{3}^{2-}$ ) on the anodic H_2_O_2_ formation has been studied recently;[^10^, ^11^] (ii) to allow comparison with data in literature on flow cell anodic H_2_O_2_ formation, since the most commonly used concentration of ${\mathrm{CO}}_{3}^{2-}$ is 2 M.[^7^, ^8^, ^15^, ^16^] The electrolyte volume was varied for the different experiments. For the recirculated and single‐pass anolyte flow experiments, a peristaltic pump (VWR PP 2202) or a syringe pump (New Era NE‐4000) were used. The cathodic compartment was continuously purged with He (10 cm^3^ min^−1^) to remove the formed H_2_. A Biologic VSP potentiostat and a Voltcraft PPS‐11603 power supply were applied as power sources. In some cases, besides the overall cell voltage values (U_cell_) the anode potential values (E_anode_) were also measured by inserting a reference electrode into the anolyte inlet (Figure S2). The measured potentials were converted to the reversible hydrogen electrode (RHE) scale: E_anode_=E_(Hg/HgO)_+0.059×pH+$E_{\mathrm{Hg}/\mathrm{HgO}}^{0}$ (Hg/HgO in 1 M KOH, $E_{\mathrm{Hg}/\mathrm{HgO}}^{0}$ =0.1 V vs. RHE). The presented E_anode_ values are iR corrected (ohmic drop correction was performed using the current interrupt method).

### 2.5 Quantification of H_2_O_2_

The determination of the amount of H_2_O_2_ formed at the anode was performed through the addition of 1950 μl of 3 mM TiOSO_4_ in 3 M H_2_SO_4_ to 50 μl of anolyte sampled after the electrolysis experiment.[^10^, ^27^] This results in the following reactions:
(1)

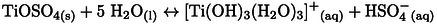



(2)

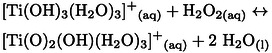




[Ti(O)_2_(OH)(H_2_O)_3_]^+^ has a yellow color, with a peak maximum in its UV‐Vis absorption spectrum at around 400 nm. By UV‐Vis spectroscopy and using calibration curves, the amount of H_2_O_2_ formed can be calculated from the determined [Ti(O)_2_(OH)(H_2_O)_3_]^+^ concentration. The H_2_O_2_ formation Faradaic efficiency (${\mathrm{FE}}_{H_{2}O_{2}}$ ) was calculated as follows:

(3) $$\mathrm{FE}=\;\frac{\;n_{H_{2}O_{2}}\;\times \;z\;\times \;F}{q}\;\times 100$$

$n_{H_{2}O_{2}}$ is the amount of moles of H_2_O_2_ formed, z is the moles of electrons required to produce 1 mole of H_2_O_2_ from H_2_O (i. e. z=2), F is the Faraday constant (i. e. F=96485 C mol^‐1^) and q the charge passed through the electrode in C.

A Shimadzu UV‐1800 UV‐Vis spectrophotometer was used for the quantification of the H_2_O_2_ concentration. The spectrophotometer was calibrated with mixtures of 50 μl 2 M K_2_CO_3_ containing different concentrations of H_2_O_2_ (0 mM, 0.78125 mM, 1.5625 mM, 3.125 mM, 6.25 mM, 12.5 mM, 25 mM, 50 mM and 100 mM) and 1950 μl of 3 mM TiOSO_4_ in 3 M H_2_SO_4_. The absorbance was measured in the range of 350 to 450 nm (Figure S3).

## 3 Results and Discussion

### 3.1 Boron‐Doped Diamond Anode

The H_2_O_2_ formation catalyst used in the present study is a boron‐doped diamond (BDD) layer coated on a Nb metal mesh.

**Figure 1:**
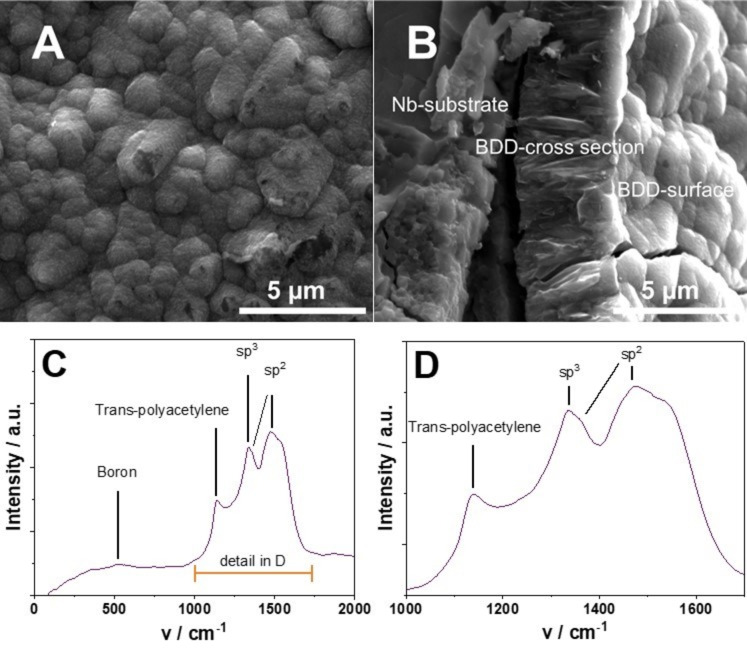
SEM micrographs of the nanocrystalline boron‐doped diamond coating. (A) top‐view, (B) cross section. (C) Raman spectra of the nanocrystalline boron‐doped diamond with the detailed carbon region in (D).

The obtained boron‐doped diamond coating has a nanocrystalline morphology, as shown in the top‐view SEM micrograph in Figure 1A, forming the typical round‐shaped agglomerations and a high density of grain boundaries. This type of CVD diamond is produced with an excess of sp3‐species and a lower amount of hydrogen radicals in the BDD deposition chamber. Under these conditions, the secondary nucleation is strongly enhanced, and diamond crystallites are nucleated at a high rate, leading finally to a coating with a crystallite size in the nanometer range. The tilt angle SEM image in Figure 1B, shows, from left to right, the metallic Nb substrate, the cross‐section of the BDD layer with a thickness of ~4 μm and the BDD top surface. Glow‐discharge optical emission spectroscopy (GDOES) measurements indicate a boron doping in the layer of 1600 ppm. The Raman spectrum in Figure 1C confirms the SEM and GDOES findings. The typical boron signature around 500 cm^−1^ is weak whereas the peaks for trans‐polyacetylene (TPA) at 1100 cm^−1^, the D‐band at 1350 cm^−1^ and the G‐band at 1500 cm^−1^ are intense and confirm the nanocrystalline nature of the diamond layer – in fact, it is known that grain boundaries are composed of non‐diamond‐carbon, i. e., sp2 carbon, and other species like trans‐polyacetylene. The D‐band is particularly prominent, and the diamond signal with which it overlaps at 1332 cm^−1^ is barely visible, and only a small shoulder can be identified.^[28]^

BDD electrodes have already proven electrochemically stable in various studies, even under elevated potentials or current densities,[^14^, ^23^] hence we do not expect significant changes in our BDD mesh electrode under the experimental conditions adopted in the present work.

### 3.2 Water Overoxidation (Competitive Oxygen Evolution Reaction)

To test the cell with the BDD mesh anode for H_2_O_2_ anodic electrosynthesis, we performed preliminary experiments under galvanostatic conditions and by continuous recirculation of the anolyte (12 cm^3^) in the anode compartment. This results in (quasi−)batch operation, similar to experimental conditions reported in previous studies in literature.[^16^, ^22^] Three different anolyte flow rates were applied ($\dot{V}$_anolyte_=24, 12 and 6 cm^3^ min^−1^, respectively). The current density was set at a constant value of 25 mA cm^−2^ (geometric area, see cell voltages in Figure S4), and the experiments had different durations (1, 2, 5, 10, 20 and 40 minutes) for all three $\dot{V}$_anolyte_ tested.

**Figure 2:**
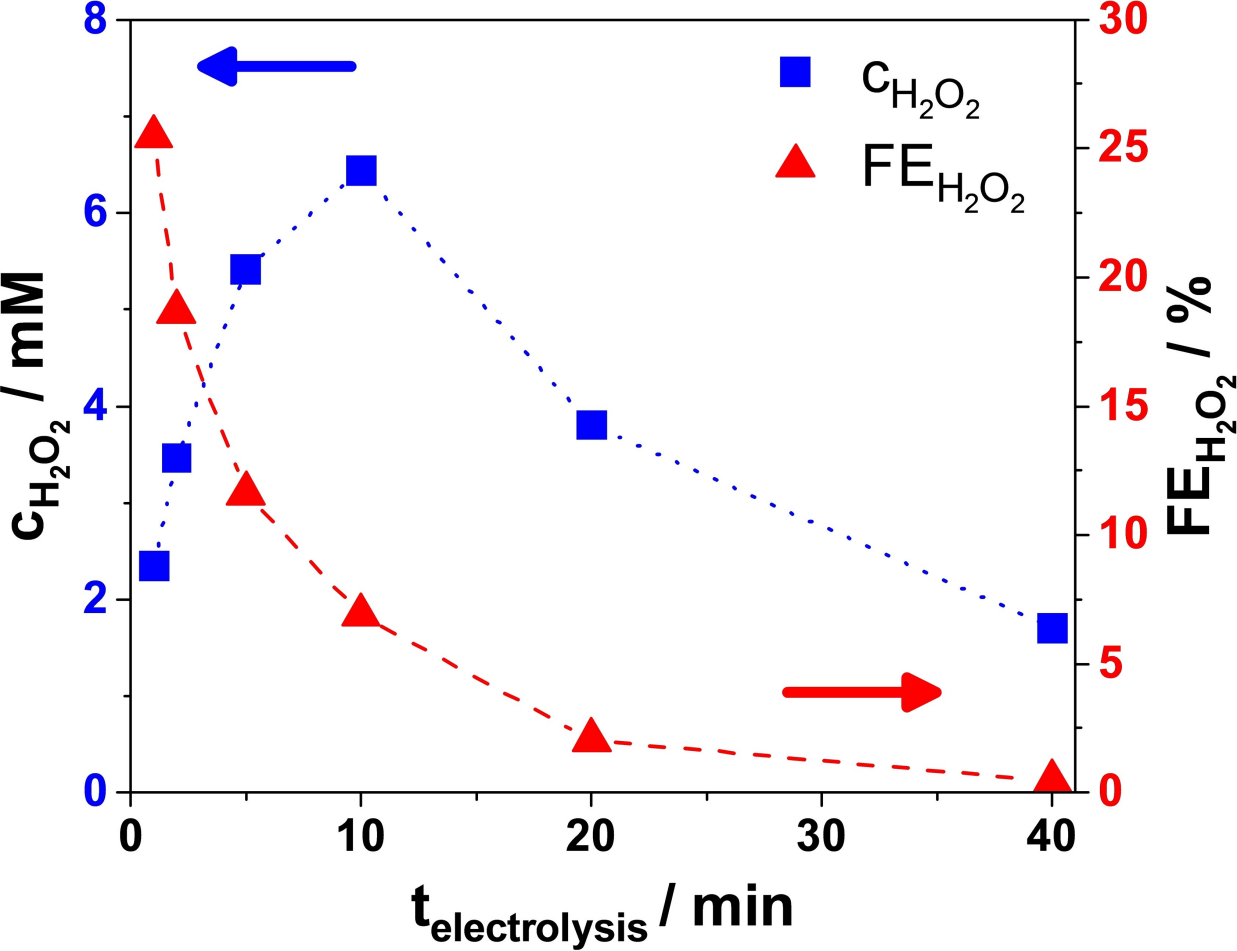
Cumulative concentration of produced H_2_O_2_ and Faradaic efficiency to H_2_O_2_ as a function of electrolysis duration (1, 2, 5, 10, 20, and 40 min) with recirculated anolyte (flow rate=24 cm^3^ min^−1^). 2 M aqueous K_2_CO_3_; V_anolyte_=12 cm^3^; j_constant_=25 mA cm^−2^.

Regardless of $\dot{V}$_anolyte_, the concentration of produced H_2_O_2_ ($c_{H_{2}O_{2}}$ , accumulated during and measured after the electrolysis) increased over time until 10 minutes of electrolysis and started to decrease with longer electrolysis times (Figure 2 and Figure S5). Moreover, the ${\mathrm{FE}}_{H_{2}O_{2}}$ dropped drastically, from 25 % to 0.5 % (Figure 2) – this is because the longer the duration of the electrolysis the more charge is passed (see Figure S6). As also supported by the literature, the reason for the decrease in $c_{H_{2}O_{2}}$ is twofold:

i) Over‐oxidation, i. e., the electrochemical oxidation of H_2_O_2_ to O_2_ takes place. This occurs because the standard potential for anodic H_2_O_2_ formation (by partial water oxidation) is more positive than that for the oxidation of H_2_O_2_ (to O_2_) according to equations (3) and (4). As $c_{H_{2}O_{2}}$ reaches a certain concentration in the electrolyte, the rate of H_2_O_2_ oxidation increases. This alone would result in a plateau of $c_{H_{2}O_{2}}$ .[^13^, ^14^]
(3)

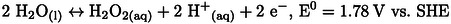



(4)

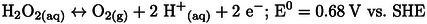




ii) The produced H_2_O_2_ undergoes spontaneous disproportionation over time (e. g., induced contact with materials such as inorganic salts, metal oxides) in the absence of any stabilizing agent (e. g. Na_2_SiO_3_) in the anolyte.[^7^, ^16^] This explains why $c_{H_{2}O_{2}}$ starts to decrease after reaching a certain concentration.[^10^, ^11^]

### 3.3 Single‐Pass Anolyte Flow

**Figure 3:**
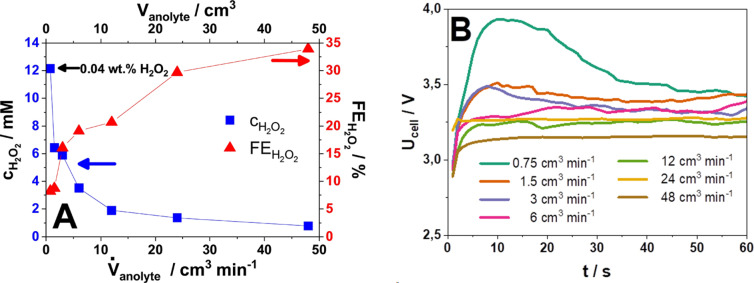
(A) Concentrations of produced H_2_O_2_ and the Faradaic efficiencies of the H_2_O_2_ formation as a function of single‐pass anolyte flow rate (0.75, 1.5, 3, 6, 12, 24, 48 cm^3^ min^−1^ respectively). (B) Cell voltages of H_2_O_2_ formation electrolysis applying constant current (j_constant_=25 mA cm^−2^) and single‐pass anolyte with different flow rates (0.75, 1.5, 3, 6, 12, 24 or 48 cm^3^ min^−1^ respectively). 2 M K_2_CO_3_; V_anolyte_=0.75, 1.5, 3, 6, 12, 24, 48 cm^3^ respectively; j_constant_=25 mA cm^−2^; 1 min electrolysis.

As these results clearly prove the limitation of a batch cell configuration, we investigated the effect of a single‐pass anolyte flow approach (to avoid water over‐oxidation) with shorter electrolysis durations (to avoid H_2_O_2_ disproportionation in the absence of a stabilizer) on the resulting $c_{H_{2}O_{2}}$ and ${\mathrm{FE}}_{H_{2}O_{2}}$ .We performed electrolysis experiments by systematically varying the anolyte flow rate and volume: $\dot{V}$_anolyte_=0.75, 1.5, 3, 6, 12, 24, and 48 cm^3^ min^‐1^. The duration and the applied constant current density were the same for each electrolysis (1 min and 25 mA cm^−2^ respectively, resulting q=21.66 C). We observed that ${\mathrm{FE}}_{H_{2}O_{2}}$ (and $n_{H_{2}O_{2}}$ ) increases by increasing $\dot{V}$_anolyte_ whereas $c_{H_{2}O_{2}}$ decreases following an opposite trend due to the more diluted H_2_O_2_ product stream. (Figure 3A and Figure S7). Results confirm that a higher anolyte flow rate allows to remove the product (H_2_O_2_), hence limiting its further oxidation, and at the same time supply fresh electrolyte to the electrode surface (hence limiting, for example, local pH changes that could affect the selectivity). This is in good agreement with the results described in the literature.^[16]^

Interestingly, U_cell_ values consistently decrease with increasing $\dot{V}$_anolyte_ (Figure 3B). We propose multiple reasons to explain this effect:

- Formation of O_2_ bubbles (resulting from water over‐oxidation, i. e., the competing oxygen evolution reaction, OER) and their detachment from the electrode is more efficient at high anolyte flow rates. Removing bubbles, which would otherwise block the electrode surface, decreases the cell resistance and cell voltage.^[29]^
- The supply of (fresh) electrolyte is faster in the case of higher $\dot{V}$_anolyte_, and this can also help to keep the cell voltage low, by limiting concentration (Nernstian) overpotential effects.

Moreover, increasing the anolyte flow rate helps to replenish the surface of the electrode with fresh electrolyte, maintain a constant interfacial pH, and transport away the products. Particularly, increasing the anolyte flow rate helps to remove the produced H_2_O_2_ from the surface of the electrode, which reduces the extent of electrochemical oxidation of H_2_O_2_ to O_2_.

However, exploring the exact reason – for the trend of cell voltage as a function of the anolyte flow rate – is beyond the scope of this article.

Since the industrially relevant concentration of H_2_O_2_ is at least 3 wt.% (typical concentration for household products used as disinfectant) ($c_{H_{2}O_{2}}$ ≈880 mM), additional improvements in process operation are necessary.^[16]^

### 3.4 Pulsed Operation

An alternative to increasing the liquid flow rate to reduce the exposure time of newly formed H_2_O_2_ to the BDD electrode, is to temporarily reduce the applied current to near zero to prevent further oxidation of H_2_O_2_. Pulsed electrolysis has been recently studied for different electrolysis applications.[^30^, ^31^, ^32^, ^33^, ^34^, ^35^] For water electrolysis, by applying pulsed voltage, lower energy demand for hydrogen production was achieved combined with electrode and membrane surface corrosion mitigation compared to electrolysis at constant current conditions;^[35]^ and for CO_2_ reduction, pulsed electrolysis can increase selectivity towards certain products.^[30]^ Previous authors obtained higher H_2_O_2_ formation rates by applying pulsed current for the oxygen reduction reaction instead of constant current: the formed H_2_O_2_ could diffuse away from the electrode surface and cathodic decomposition of H_2_O_2_ was avoided.^[36]^

**Figure 4:**
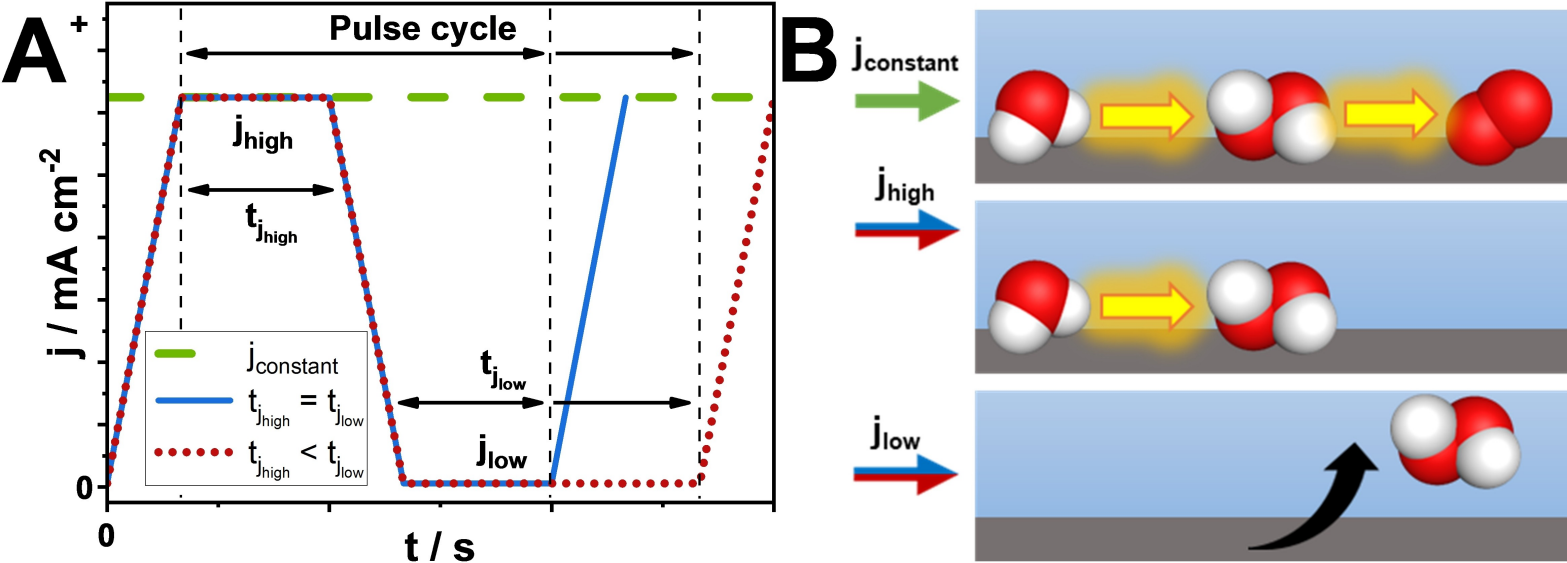
(A) Sketch of a pulse cycle during pulsed current electrolysis, and (B) expected effect of constant vs. pulsed current electrolysis on $c_{H_{2}O_{2}}$ and ${\mathrm{FE}}_{H_{2}O_{2}}$ as a function of the current‐pulse shape (j_high_ vs. j_low_, $t_{j_{\mathrm{high}}}$ vs. $t_{j_{\mathrm{low}}}$ ).

To achieve a high anodically produced $c_{H_{2}O_{2}}$ with low $\dot{V}$_anolyte_, we investigated the effect of pulsed current on the ${\mathrm{FE}}_{H_{2}O_{2}}$ . The pulse frequency we have chosen is based on ref.^[36]^ We started from such pulse frequency and varied it systematically to investigate the effects on our system. Our results prove that the pulse frequencies we chose fall in a relevant range, as significant differences in FE could be obtained. In further work, modelling could provide more predictive insights on the correlation between pulse frequency and flow rate. In the present study, a $\dot{V}$_anolyte_ of 0.75 cm^3^ min^−1^ was selected. During the pulsed electrolysis experiments, we applied a current density (j_high_) for the formation of the H_2_O_2_ and a current density (j_low_) at which no significant amounts of H_2_O_2_ would be formed. In addition, we systematically changed the ratio between the durations of j_high_ and j_low_, to determine the impact of the off‐time on the ${\mathrm{FE}}_{H_{2}O_{2}}$ and $c_{H_{2}O_{2}}$ . To provide enough time for the removal of produced H_2_O_2_ from the electrode surface, different current densities (j_high_ and j_low_) were applied alternatingely for certain periods of time ($j_{\mathrm{high}}$ and $t_{j_{\mathrm{low}}}$ ). This resulted in a pulse cycle (full cycle duration = $t_{j_{\mathrm{high}}}$ +$t_{j_{\mathrm{low}}}$ ) with certain amplitude j_high_‐j_low_ and ratio of $t_{j_{\mathrm{high}}}$ to $t_{j_{\mathrm{low}}}$ (see Figure 4). Zero current (j=0 mA cm^−2^) was not applied as j_low_ in order to avoid the cell voltage reaching open circuit conditions (quasi‐shutdown), since it is known from fuel cell and water electrolysis literature that this can have negative effects on the device stability.[^37^, ^38^] The j_high_ value was the same in every pulsed experiments and it was equal to that used during constant current experiments (25 mA cm^−2^), meanwhile j_low_ was set to 1 % of j_high_ (i. e., 0.25 mA cm^−2^), unless otherwise indicated. The duration of the electrolysis experiments was 5 minutes. Below, we use the results of constant current electrolysis experiments as reference.

#### 3.4.1 Equal tjhigh and tjlow Durations

**Figure 5:**
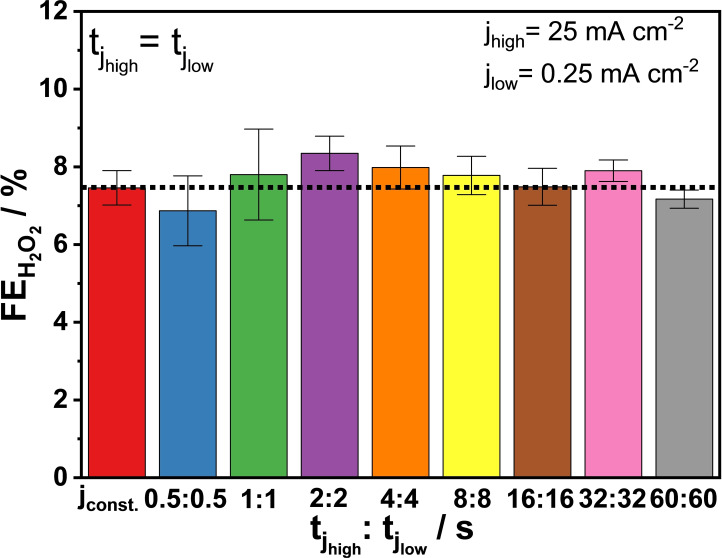
Faradaic efficiencies for H_2_O_2_ formation at constant current (j_constant_=25 mA cm^−2^) or pulsed current with different $t_{j_{\mathrm{high}}}$ /$t_{j_{\mathrm{low}}}$ ratio: 0.5 s/0.5 s, 1 s/1 s, 2 s/2 s, 4 s/4 s, 8 s/8 s, 16 s/16 s, 32 s/32 s, and 60 s/60 s. j_high_=25 mA cm^−2^, j_low_=0.25 mA cm^−2^; 2 M K_2_CO_3_; V_anolyte_=4.5 cm^3^; single‐pass anolyte, 0.75 cm^3^ min^−1^; 5 min electrolysis. Error bars are derived from the results of 3 identical experiments for each experimental condition.

In a first series of experiments, $t_{j_{\mathrm{high}}}$ and $t_{j_{\mathrm{low}}}$ had the same duration within a current‐pulse cycle. The length of each pulse cycle was systematically increased (f_pulse_=1 Hz to 8 mHz), consistent with experimental conditions found in the literature (see the current profiles in Figure S8).^[36]^ The change of the measured cell voltage followed the periodical change of the applied current density and fluctuated between 1.7 and 3.3 V for j_low_ and j_high_, respectively (Figure S9). The resulting ${\mathrm{FE}}_{H_{2}O_{2}}$ values for the pulsed electrolysis experiments did not differ much among each other, and from the reference experiment at constant current (j_constant_) (Figure 5). The 1 : 1 ratio of $t_{j_{\mathrm{high}}}$ and $t_{j_{\mathrm{low}}}$ investigated, thus, seems to not have any significant effect on the ${\mathrm{FE}}_{H_{2}O_{2}}$ . This can be explained as:

- the overall $t_{j_{\mathrm{high}}}$ and $t_{j_{\mathrm{low}}}$ ($\Sigma t_{j_{\mathrm{high}}}$ and ${\Sigma t}_{j_{\mathrm{low}}}$ ) were equal for the experiments;
- the total amount of charge passed during the j_low_ and j_high_ phase (${\Sigma q}_{j_{\mathrm{low}}}$ and ${\Sigma q}_{j_{\mathrm{high}}}$ ) was equal for the experiments;
- the ratio of the amount of charge passed during the j_low_ and j_high_ phase (${\Sigma q}_{j_{\mathrm{low}}}$ /${\Sigma q}_{j_{\mathrm{high}}}$ ) was equal for the experiments;
- the total amount of charge passed during the experiments (Σq) was also almost identical (Figure S10A).

Although these experiments were performed by applying pulsed current, the chosen equal durations for j_high_ and j_low_ resulted in a quasi‐constant current electrolysis for half of the duration of the experiments. We therefore wanted to see if there is an effect of on‐ and off‐phases with different durations, and if there is an optimal ratio between these durations – that is, an optimal on‐time for the formation of H_2_O_2_, and an optimal off‐time to enable mass transport.

#### 3.4.2 Varied tjhigh and tjlow Durations

**Figure 6:**
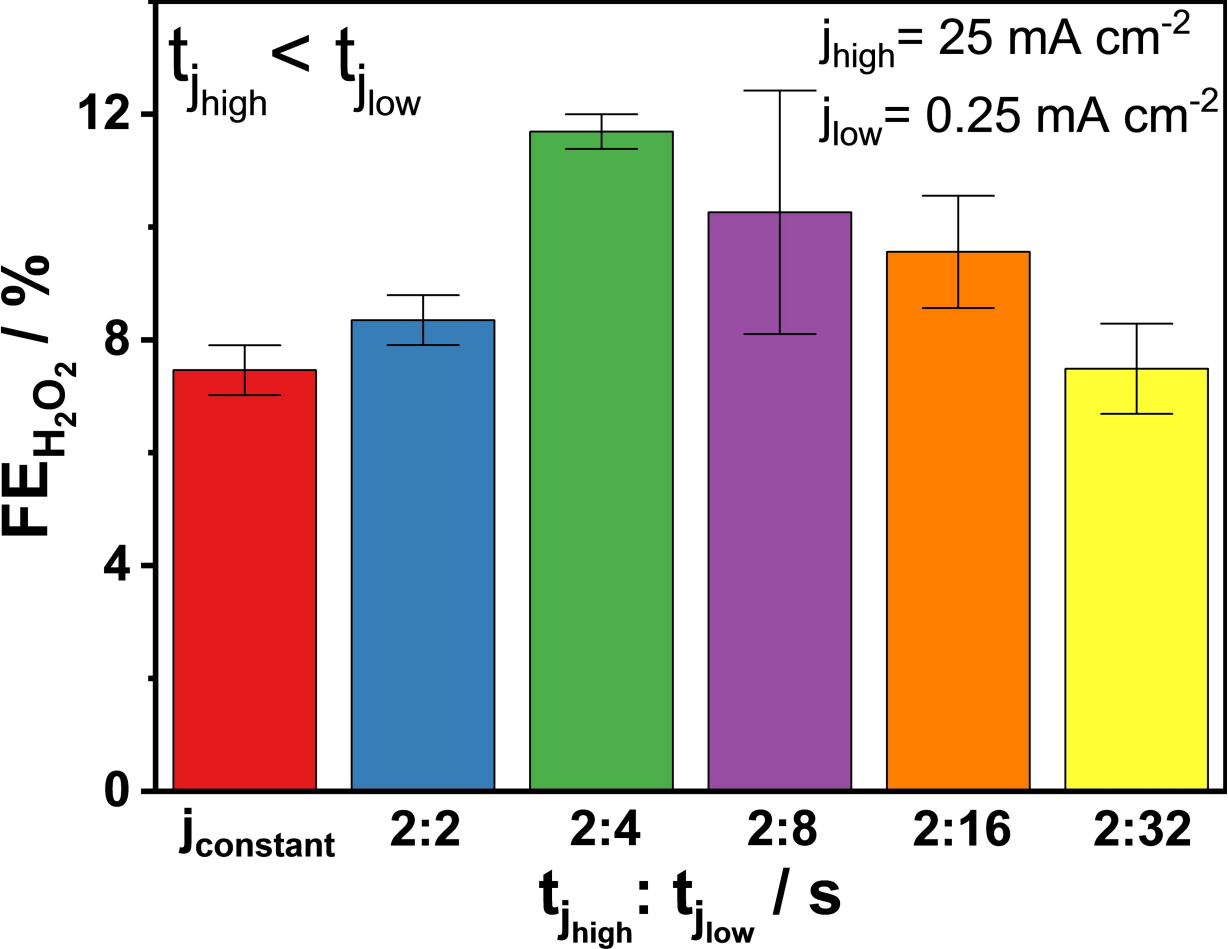
Faradaic efficiencies for H_2_O_2_ formation at constant current (j_constant_=25 mA cm^−2^) or pulsed current with different $t_{j_{\mathrm{high}}}$ /$t_{j_{\mathrm{low}}}$ ratio: $t_{j_{\mathrm{high}}}$ =2 s; $t_{j_{\mathrm{low}}}$ =2 s, 4 s, 8 s, 16 s, and 32 s. j_high_=25 mA cm^−2^, j_low_=0.25 mA cm^−2^; 2 M K_2_CO_3_; V_anolyte_=4.5 cm^3^; single‐pass anolyte, 0.75 cm^3^ min^−1^; 5 min electrolysis. Error bars are derived from results of 3 identical experiments for each experimental condition.

In the following experiments, the $t_{j_{\mathrm{high}}}$ /$t_{j_{\mathrm{low}}}$ ratio was modified: $t_{j_{\mathrm{high}}}$ was kept constant (2 s) and $t_{j_{\mathrm{low}}}$ was systematically varied (2, 4, 8, 16 and 32 s, see Figure S11) in order to obtain different $\Sigma t_{j_{\mathrm{high}}}$ and ${\Sigma t}_{j_{\mathrm{low}}}$ , resulting in different $\Sigma t_{j_{\mathrm{high}}}$ /${\Sigma t}_{j_{\mathrm{low}}}$ and ${\Sigma q}_{j_{\mathrm{low}}}$ /${\Sigma q}_{j_{high}}$ ratios, and to obtain different Σq values (Figure S13A). In the case of the 2 s j_high_/4 s j_low_ experiment, ${\mathrm{FE}}_{H_{2}O_{2}}$ was highest: significantly higher than in the case of the constant current and the other pulsed electrolysis experiments (Figure 6). To explain these results, we propose the following reasons:

- The formed H_2_O_2_ is pushed away from the electrode (i. e., from the reactive area near the electrode/electrolyte interface) and hence it is not further oxidized electrochemically.
- The electrode surface is being replenished with fresh electrolyte. As the undesired WOR leads to a local acidification of the ${\mathrm{CO}}_{3}^{2-}$ electrolyte at the electrode/electrolyte interface, there is a shift of the ${\mathrm{CO}}_{3}^{2-}$ equilibrium towards ${H\mathrm{CO}}_{3}^{-}$ and CO_2_ which reduces the local concentration of ${\mathrm{CO}}_{3}^{2-}$ and in turn affects the selectivity. By refreshing the anolyte, the local alkaline pH and ${\mathrm{CO}}_{3}^{2-}$ concentration can be kept constant to sustain the generation of ${\mathrm{CO}}_{3}^{•-}$ radicals for H_2_O_2_ formation. 4 s j_low_ seems to be enough time for the pH to equilibrate with the applied flow conditions, 0.75 cm^3^ min^−1^.

The role of varying current density on the balance between H₂O₂ production and over‐oxidation was not assessed directly. However, both datasets in Figure 6 and Figure 9 (discussed later) indicate that under constant current conditions the ${\mathrm{FE}}_{H_{2}O_{2}}$ drops with increasing charge passed, while pulsed operation alleviates the issue of overoxidation to a significant extent . For both current density values in Figure 6 and Figure 9 (25 and 150 mA cm^−2^, respectively), the increase of ${\mathrm{FE}}_{H_{2}O_{2}}$ is similar (1.6 and 1.7 times higher, respectively) when comparing pulsed vs. constant current electrolysis. Hence, for both high and low current density regimes, the problem of constant current operation is that electrochemical oxidation of the formed H_2_O_2_ takes place considerably.

If for electrolysis experiments performed under the same conditions (duration, temperature, etc.) the chemical decomposition of H_2_O_2_ occurs to a comparable extent (same amount of H_2_O_2_ chemically oxidized to O_2_), we can estimate the amount of H_2_O_2_ that is electrochemically oxidized to O_2_ if constant current is applied instead of pulsed current, using data in Figure 6 and Figure S13A and B. Based on the calculations provided in the SI, about 1/3 of the amount of H_2_O_2_ produced is being electrochemically oxidized, if constant current electrolysis is applied instead of pulsed current electrolysis.

We also noticed that the ${\mathrm{FE}}_{H_{2}O_{2}}$ drops if we increase $t_{j_{\mathrm{low}}}$ further (>4 s) and the reason for this has still to be identified. To fully understand the latter and, more importantly, the origin of the performance increase upon optimizing the current pulse profile, multiscale modelling and operando *in‐situ* spectro‐electrochemical studies are needed.

In the following experiments, we kept using the 2 s j_high_/4 s j_low_ pulse (f_pulse_=167 mHz). Based on current density values used in experiment discussed above, ${\Sigma q}_{j_{\mathrm{low}}}$ (total charge passed at j_low_=0.25 mA cm^−2^) is min. 1 % and max. 16 % of ${\Sigma q}_{j_{\mathrm{high}}}$ (total charge passed at j_high_=25 mA cm^−2^), depending on the $t_{j_{\mathrm{high}}}$ /$t_{j_{\mathrm{low}}}$ ratio. We explored further the effect of varying j_low_, and three j_low_ values were examined: 2.5 mA cm^−2^, 0.25 mA cm^−2^ and 0.025 mA cm^−2^ (Figure S14). j_high_ was the same as in previous experiments (25 mA cm^−2^).

**Figure 7:**
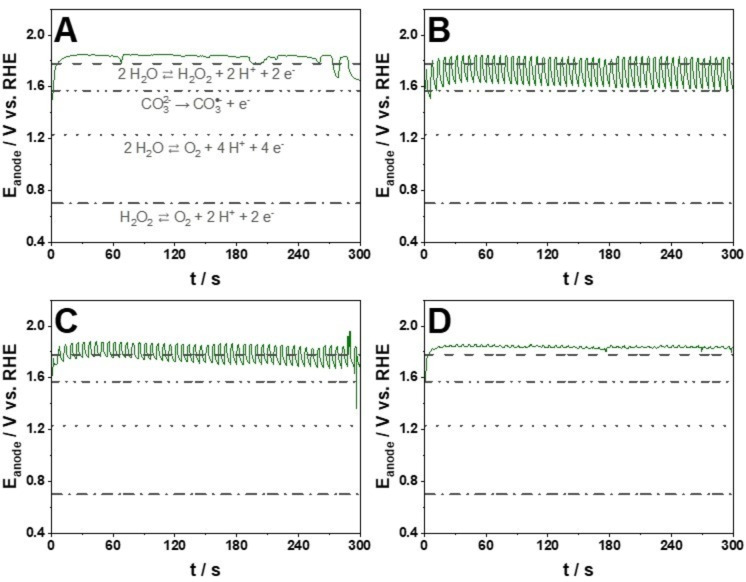
Anode potential values during H_2_O_2_ formation electrolysis at constant current (j_constant_=25 mA cm^−2^ (A)) or pulsed current with different pulse amplitudes: $t_{j_{high}}$ /$t_{j_{low}}$ =2 s/4 s; j_high_=25 mA cm^−2^, j_low_=0.025 (B), 0.25 (C) or 2.5 (D) mA cm^−2^. E_anode_=E_(Hg/HgO)_+0.059×pH+$E_{\mathrm{Hg}/\mathrm{HgO}}^{0}$ (Hg/HgO in 1 M KOH, $E_{\mathrm{Hg}/\mathrm{HgO}}^{0}$ =0.1 V vs. RHE). Standard equilibrium potentials of competing reactions are indicated for reference. 2 M K_2_CO_3_; V_anolyte_=4.5 cm^3^; single‐pass anolyte, 0.75 cm^3^ min^−1^; 5 min electrolysis.

During this set of experiments, besides the cell voltage (U_cell_), the anode potential (E_anode_) was also measured using a reference electrode inserted into the anolyte inlet (Figure S2). We observed significant differences in U_cell_ and E_anode_ with varying j_low_: the lower the j_low_ value, the lower the corresponding U_cell_ (Figure S15) and E_anode_ (Figure 7). E_anode_ remained more positive than the H_2_O_2_ oxidation potential (0.68 V vs. RHE) in every experiment regardless of the j_low_ values. This might imply that undesired electrochemical oxidation of produced H_2_O_2_ took place also during the j_low_ phase of the pulse, however to a limited extent due to the significantly lower charge passed at j_low_ (Figure S16 A).

**Figure 8:**
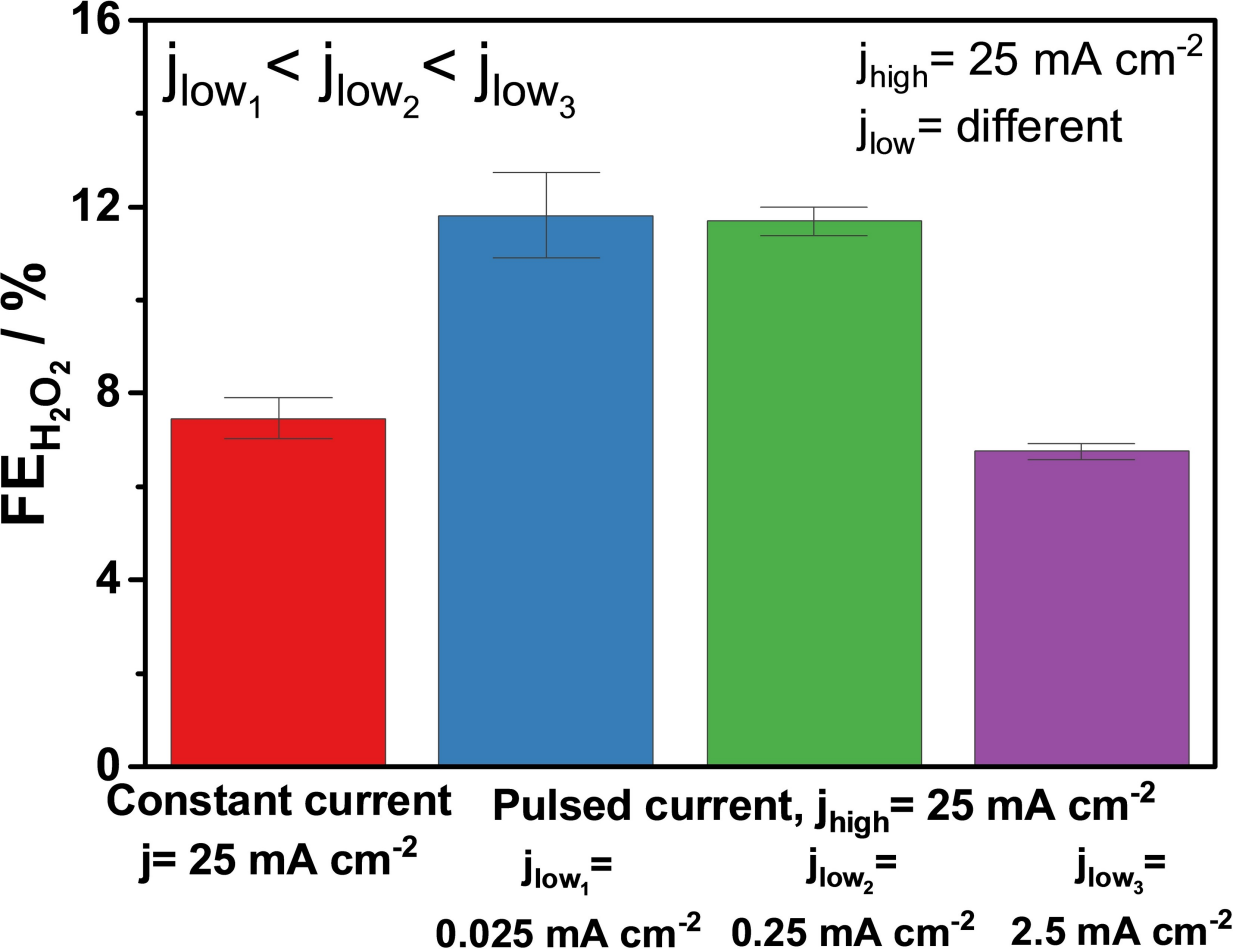
Faradaic efficiencies for H_2_O_2_ formation at constant current (j_constant_=25 mA cm^−2^) or pulsed current with different pulse amplitudes: $t_{j_{\mathrm{high}}}$ /$t_{j_{\mathrm{low}}}$ =2 s/4 s; j_high_=25 mA cm^−2^, j_low_=0.025, 0.25, and 2.5 mA cm^−2^. 2 M K_2_CO_3_; V_anolyte_=4.5 cm^3^; single‐pass anolyte, 0.75 cm^3^ min^−1^; 5 min electrolysis. Error bars are derived from results of 3 identical experiments for each experimental condition.

${\Sigma q}_{j_{\mathrm{low}}}$ was 10 %, 1 % and 0.1 % of ${\Sigma q}_{j_{\mathrm{high}}}$ for j_low_=2.5 mA cm^−2^, 0.25 mA cm^−2^ and 0.025 mA cm^−2^, respectively. The difference has evident effects on the ${\mathrm{FE}}_{H_{2}O_{2}}$ : low j_low_ values, such as 0.25 mA cm^−2^ and 0.025 mA cm^−2^, lead to higher ${\mathrm{FE}}_{H_{2}O_{2}}$ (Figure 8) compared with pulsed experiments with j_low_=2.5 mA cm^−2^ or with constant current experiments.

#### 3.4.3 Operation at Industrially Relevant Current Densities

**Figure 9:**
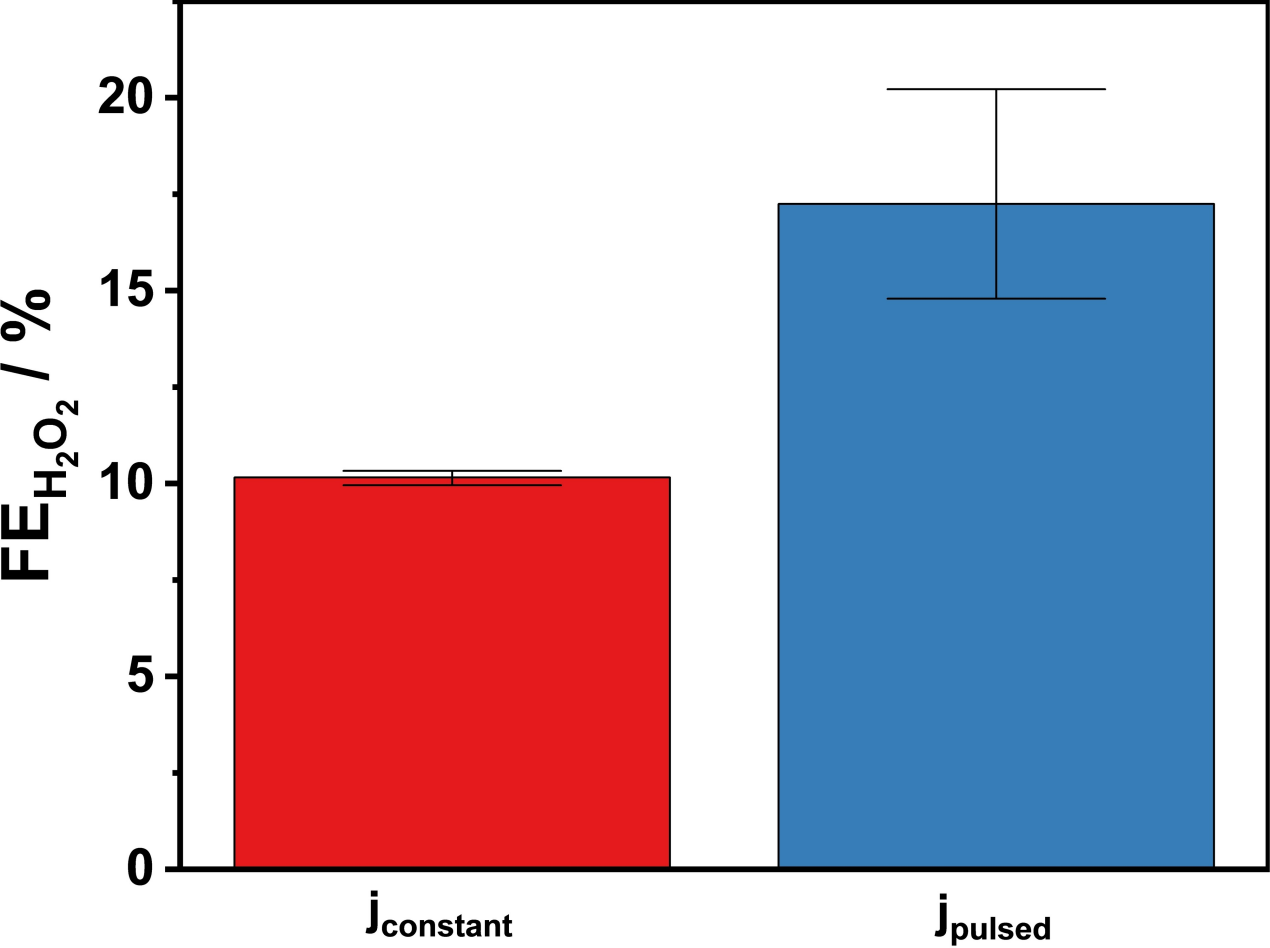
Faradaic efficiencies for H_2_O_2_ formation at constant current (j_constant_=150 mA cm^−2^) or pulsed current ($t_{j_{\mathrm{high}}}$ /$t_{j_{\mathrm{low}}}$ =2 s/4 s; j_high_=150 mA cm^−2^, j_low_=1.5 mA cm^−2^). 2 M K_2_CO_3_; V_anolyte_=4.5 cm^3^; single‐pass anolyte, 0.75 cm^3^ min^−1^; 5 min electrolysis. Error bars are derived from results of 3 identical experiments for each experimental condition.

Finally, electrolysis experiments at industrially relevant current densities were performed applying both constant (j_constant_=150 mA cm^−2^) and pulsed current (j_high_=150 mA cm^−2^ for 2 s and j_low_=1.5 mA cm^−2^ for 4 s) respectively (Figure S17 and Figure S18). The difference in the ${\mathrm{FE}}_{H_{2}O_{2}}$ between the two types of electrolysis was similar to that at lower current densities (Figure 9): by a factor of 1.7 in the favor of the pulsed electrolysis. The amount of charge passed during the constant current electrolysis was almost three times more than that of the pulsed electrolysis (Figure S19A), but the concentration of the produced H_2_O_2_ was only less than double of it (Figure S19B).

The two types of electrolysis were also compared in the terms of power efficiency (we multiplied the cell voltage (V) and the current (A) values at each recorded measurement point (every 1 s) and then averaged the obtained values (P)). We found that the power demand of the constant current electrolysis was 10.8 W, which is 2.8 times higher than that of the pulsed electrolysis 3.8 W, but only 1.7 times more H_2_O_2_ was produced. Finally, we calculated an increase in overall energy efficiency (EE) by ca. 63 % for the pulsed current operation compared to the constant current operation in terms of H_2_O_2_ production per unit energy (${\mathrm{mol}}_{H_{2}O_{2}}$ kWh^−1^, see calculation in the SI). It is expected that by optimizing electrode and cell properties and process parameters, the energy consumption can be further decreased, ideally to an economically feasible level.

### 3.5 Prospects

Our results open a large parameter space to further optimize electrolysis systems for H_2_O_2_ production. This includes: (i) the MEA components (BDD: B content, structure/morphology; polymer membranes, cathode GDE) and hardware, i. e., cell design (e. g., flow fields), to decrease the cell voltage and improve the energy efficiency; and (ii) the reaction parameters (e. g., $\dot{V}$_anolyte_, current pulse on‐time/off‐time ratio, electrolyte concentration, temperature), to maximize the ${\mathrm{FE}}_{H_{2}O_{2}}$ and $c_{H_{2}O_{2}}$ . Another interesting and important aspect is the long‐term stability of the cell as it is operated under dynamic conditions. Degradation may result from dynamic operation, such as chemical degradation of the MEA components, particularly membrane degradation issues, or mechanical degradation, since the H_2_O_2_ forming anode is pressed against the proton exchange membrane. These aspects remain to be investigated in future work.[^39^, ^40^]

## 4 Conclusions

In this study, we presented a new cell design for a zero‐gap PEM electrolyzer with a BDD‐coated Nb mesh anode for the formation of H_2_O_2_ by partial WOR; we also introduced the concept of pulsed electrolysis for the anodic formation of H_2_O_2_ in a zero‐gap PEM flow‐cell.

Optimizing $\dot{V}$_anolyte_ alone provides only limited improvement in the attempt to reach high $c_{H_{2}O_{2}}$ and ${\mathrm{FE}}_{H_{2}O_{2}}$ . Instead, combining control of the anolyte flow rate with pulsed current electrolysis (and potentially even more under discontinuous flow operation, i. e., intermittent pump operation) opens new possibilities to prevent oxidation of H_2_O_2_. In this case, the influential parameters, such as pulse profile (which affects the product formation rate) and $\dot{V}$_anolyte_ (which affects the removal of the product and the supply of reactants), were applied in an optimal way, resulting in better efficiency. Nevertheless, it is important to note that these parameters affect each other, thus by changing $\dot{V}$_anolyte_ a different f_pulse_ might become optimal. This optimization can be achieved experimentally (phenomenological approach), while modelling, if possible, would allow to narrow the parameter space for screening in order to identify optimal sets of conditions.

## Conflict of Interests

The authors declare that S.R. is a cofounder and shareholder of DiaCCon GmbH, Fuerth, Germany.

5

## Supporting information

As a service to our authors and readers, this journal provides supporting information supplied by the authors. Such materials are peer reviewed and may be re‐organized for online delivery, but are not copy‐edited or typeset. Technical support issues arising from supporting information (other than missing files) should be addressed to the authors.

Supporting Information

## Data Availability

The data that support the findings of this study are available from the corresponding author upon reasonable request.
