# Supplementary material for: Pulsed‐Current Operation Enhances H2O2 Production on a Boron‐Doped Diamond Mesh Anode in a Zero‐Gap PEM Electrolyzer
Source: ChemSusChem. 2025 Jan 16;18(9):e202401947. doi: 10.1002/cssc.202401947 (PMC12051251; doi:10.1002/cssc.202401947)
Supplement: Supplementary file 1 — Supporting Information [file CSSC-18-e202401947-s001.pdf]

ChemSusChem

Supporting Information

Pulsed-Current Operation Enhances H_2O_2 Production on a Boron-Doped Diamond Mesh Anode in a Zero-Gap PEM Electrolyzer

Adam Vass, Maximilian Göltz, Hanadi Ghanem, Stefan Rosiwal, Tanja Franken,
Regina Palkovits, Guido Mul, Mihalis N. Tsampas, Georgios Katsoukis, and Marco Altomare*

Supporting Information

Pulsed-Current Operation Enhances H_2O_2 Production on a Boron-Doped Diamond Mesh Anode in a Zero-Gap PEM Electrolyzer

Adam Vass[1], Maximilian Göltz[2], Hanadi Ghanem[2], Stefan Rosiwal[2], Tanja Franken[2], Regina Palkovits[3,4,5], Guido Mul[1], Mihalis N. Tsampas[6], Georgios Katsoukis[1], Marco Altomare[1]*

1 Department of Chemical Engineering, MESA+ Institute for Nanotechnology, Faculty of Science and Technology, University of Twente, Drienerlolaan 5, 7522 NB Enschede, The Netherlands

2 Friedrich-Alexander-Universität Erlangen-Nürnberg, Schloßplatz 4, 91054 Erlangen, Germany

3 Forschungszentrum Jülich, Institute for a Sustainable Hydrogen Economy (INW-2), Marie-Curie- Straße 5, 52428 Jülich, Germany

4 RWTH Aachen University, Institute for Technical and Macromolecular Chemistry, Worringerweg 2, 52074 Aachen, Germany

5 Max-Planck-Institute for Chemical Energy Conversion, Stiftstraße 34-36, 45470 Mülheim an der Ruhr, Germany

6 Dutch Institute for Fundamental Energy Research (DIFFER), 5612AJ, Eindhoven, The Netherlands

* Corresponding author. Email: m.altomare@utwente.nl

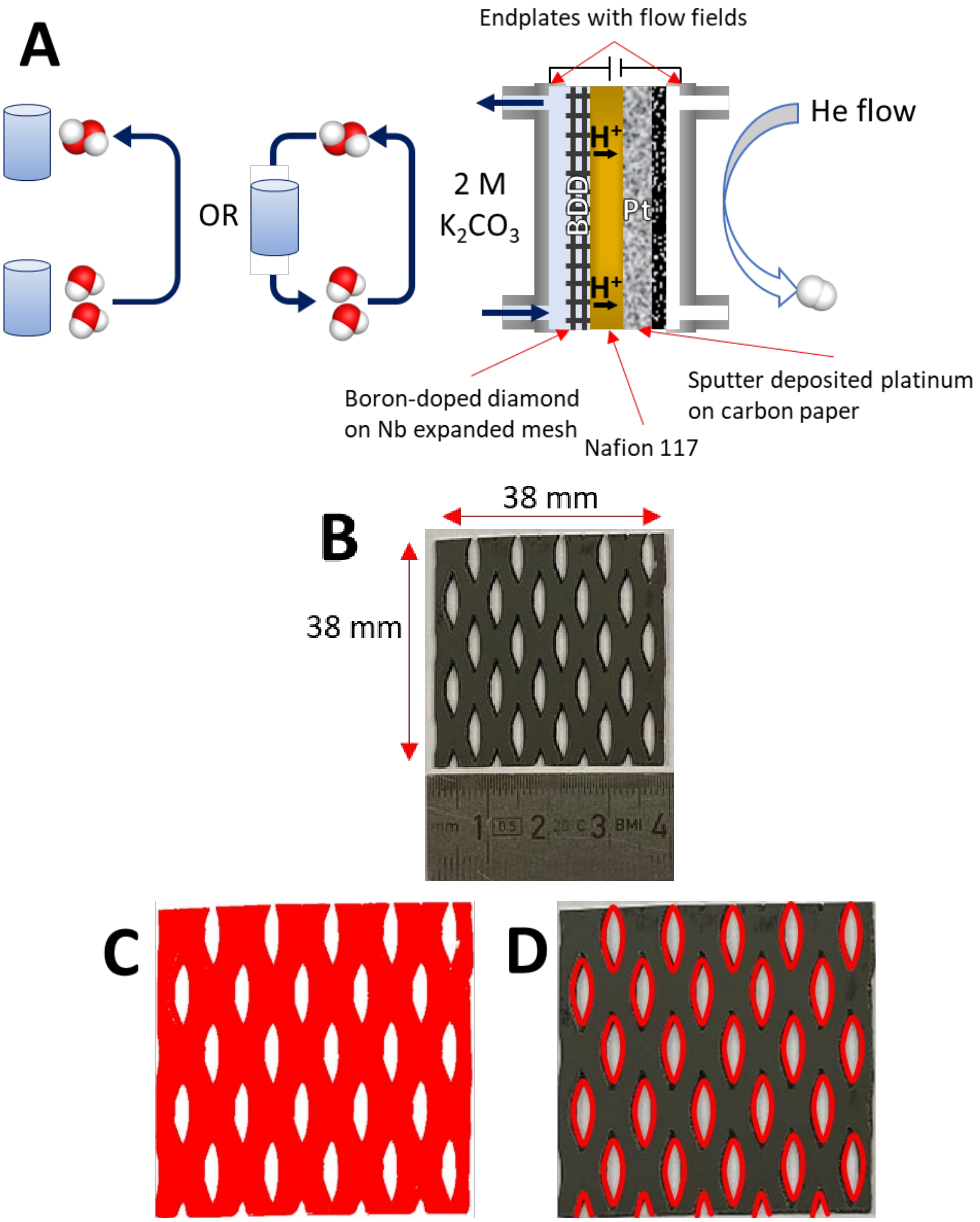

Figure S1. (A) Schematic of the cell with the schematics of recirculated and single-pass anolyte flow experiments. (B) Boron-doped diamond coated Nb expanded mesh. (C) Area of contact surface between mesh anode and membrane. (D) Sides of the mesh holes (perimeters) used for calculation.

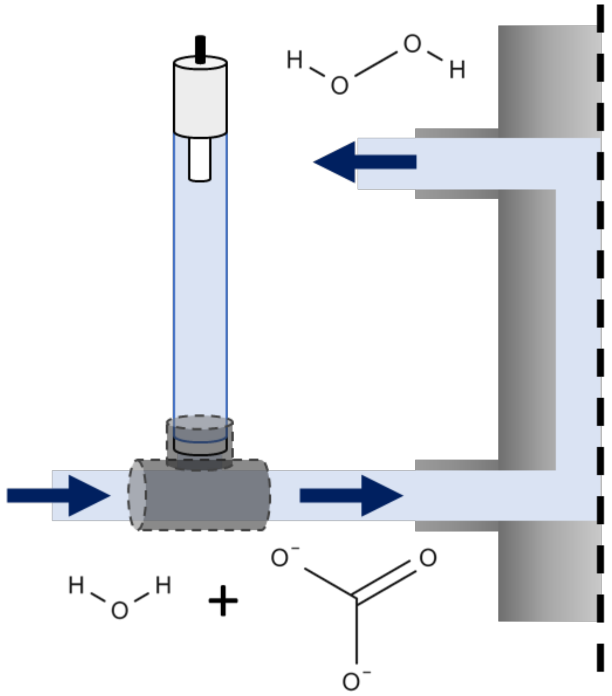

Figure S2. Schematic of inserting a reference electrode to the anolyte inlet. Hg/HgO in 1 M KOH, $E^0_{Hg/HgO}$= 0.1 V vs. RHE.

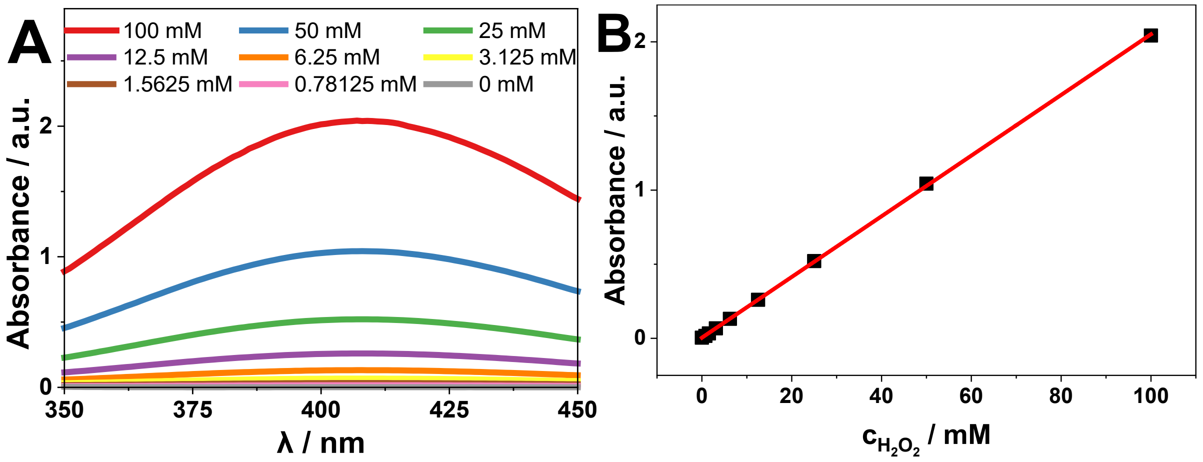

Figure S3. (A) UV-Vis absorption spectra of the mixture of 50 µl of 2 M K_2CO_3 with different concentrations of H_2O_2 (100, 50.0, 25.0, 12.5, 6.25, 3.12, 1.56, 0.78 and 0 mM) and 1950 µl of 3 mM $TiOSO_4$ in 3 M H_2SO_4 between 350 nm and 450 nm. (B) Calibration line based on the maximum values of absorbance and the H_2O_2 concentration of the sample.

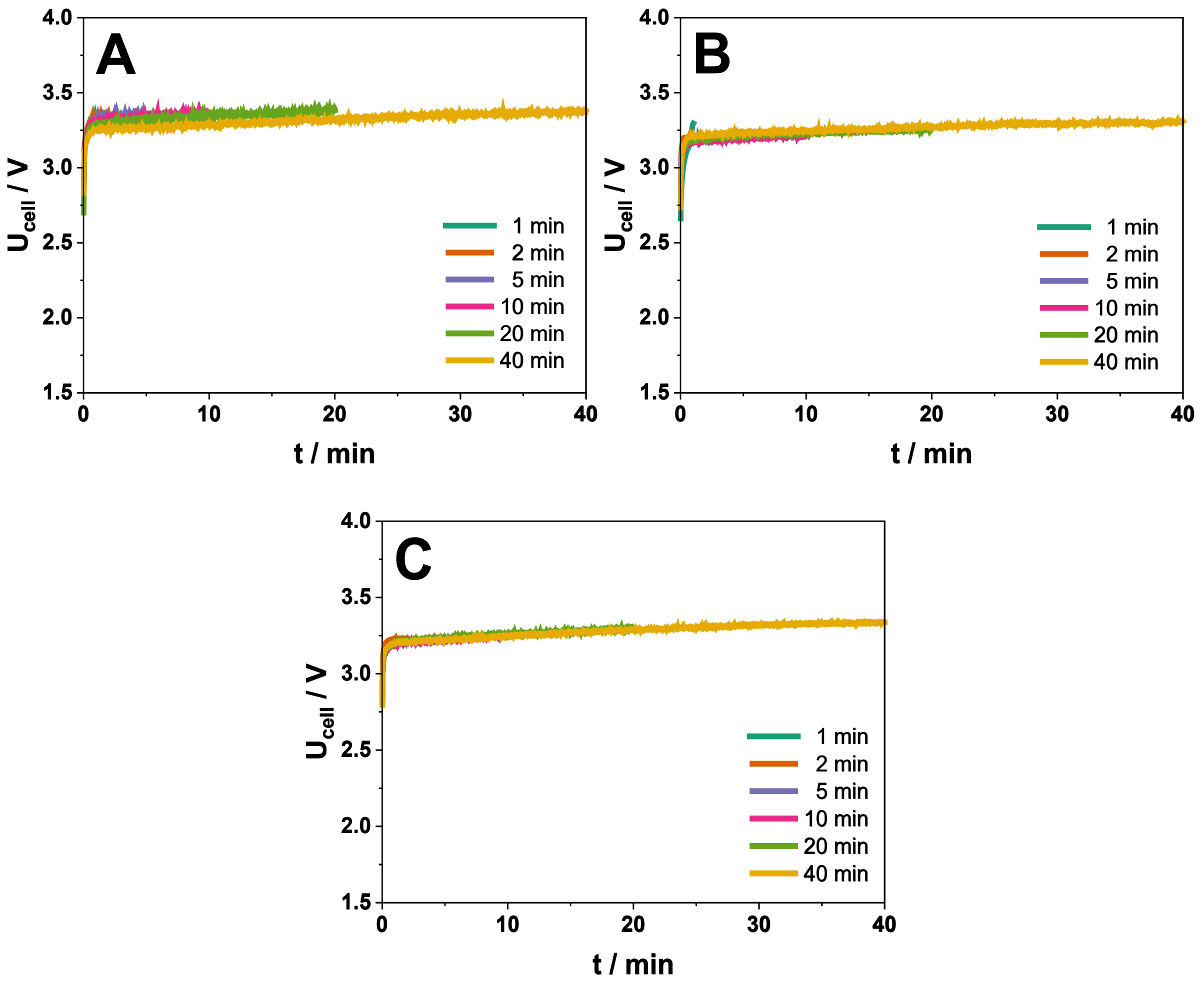

Figure S4. Measured cell voltages for H_2O_2 formation electrolysis applying constant current and recirculated anolyte with flow rates of (A) 6, (B) 12 and (C) 24 $cm^3\ min^{-1}$, as a function of time. 2 M K_2CO_3; $V_{anolyte}$= 12 cm^3; $j_{constant}$= 25 mA cm^{-2}; 1, 2 5, 10, 20 or 40 min electrolysis.

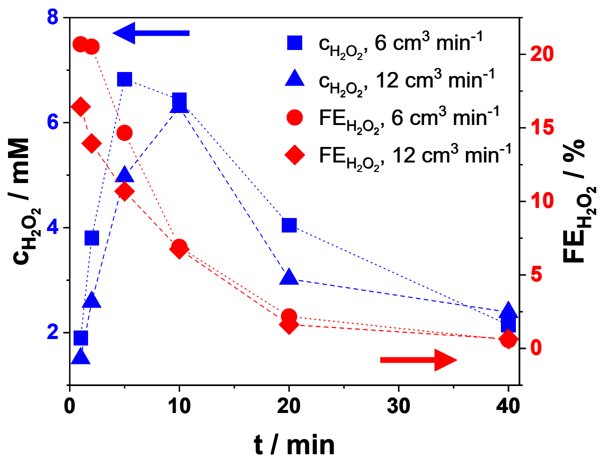

Figure S5. Cumulative concentration of produced H_2O_2 and Faradaic efficiency of the H_2O_2 formation as a function of electrolysis duration (1, 2, 5, 10, 20 or 40 min) with recirculated anolyte with flow rates of 6 or 12 $cm^3\ min^{-1}$. 2 M K_2CO_3; $V_{anolyte}$= 12 cm^3; $j_{constant}$= 25 mA cm^{-2}.

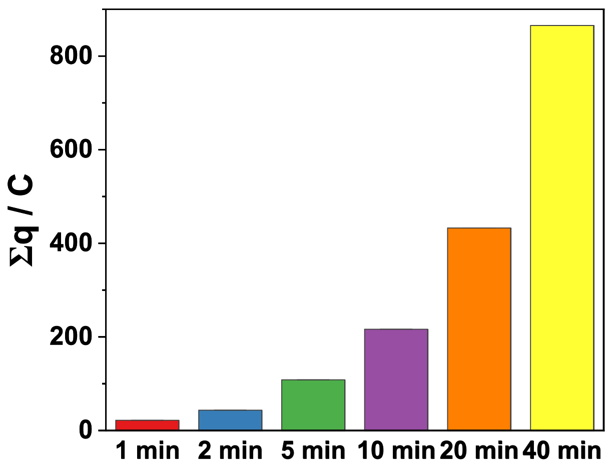

Figure S6. Quantity of charge passed during H_2O_2 formation electrolysis applying constant current ($j_{constant}$= 25 mA cm^{-2}) for different durations (1, 2, 5, 10, 20 or 40 min).

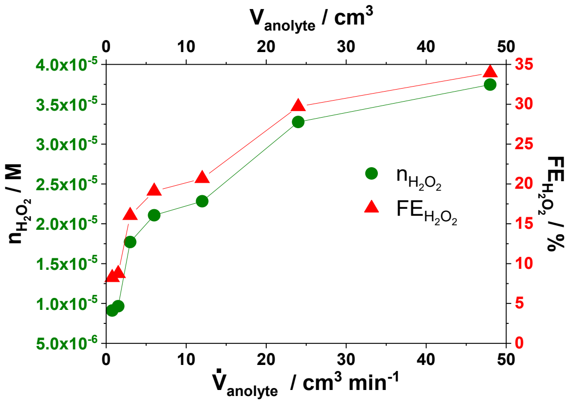

Figure S7. Amount of produced H_2O_2 and Faradaic efficiencies for H_2O_2 formation as a function of single-pass anolyte flow rate (0.75, 1.5, 3, 6, 12, 24, and 48 cm^3 min^{-1}). 2 M K_2CO_3; $V_{anolyte}$ = 0.75, 1.5, 3, 6, 12, 24, and 48 cm^3; $j_{constant}$ = 25 mA cm^{-2}; 1 min electrolysis.

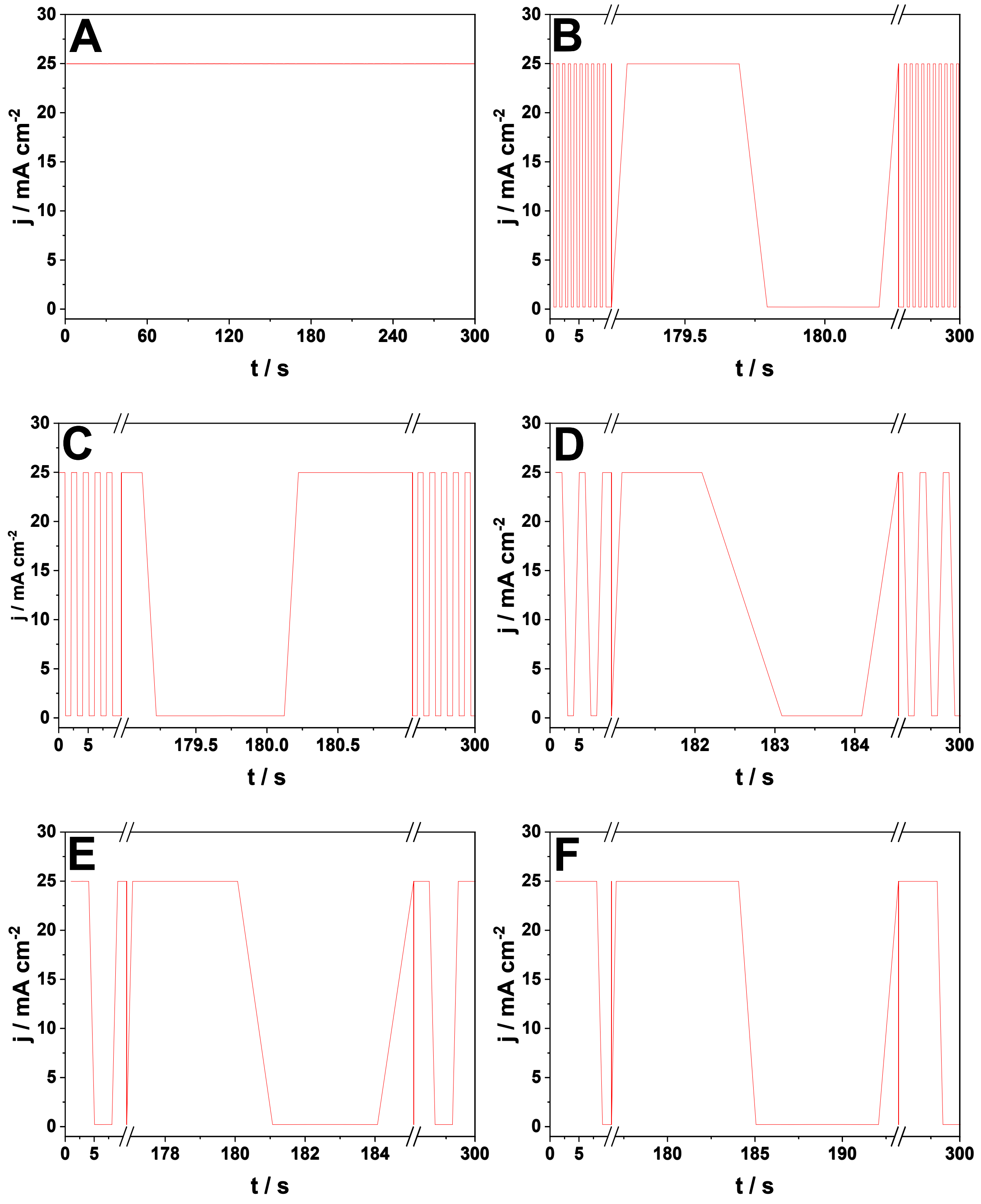

A
B
C
D
E
F
j / mA cm-2
t / s

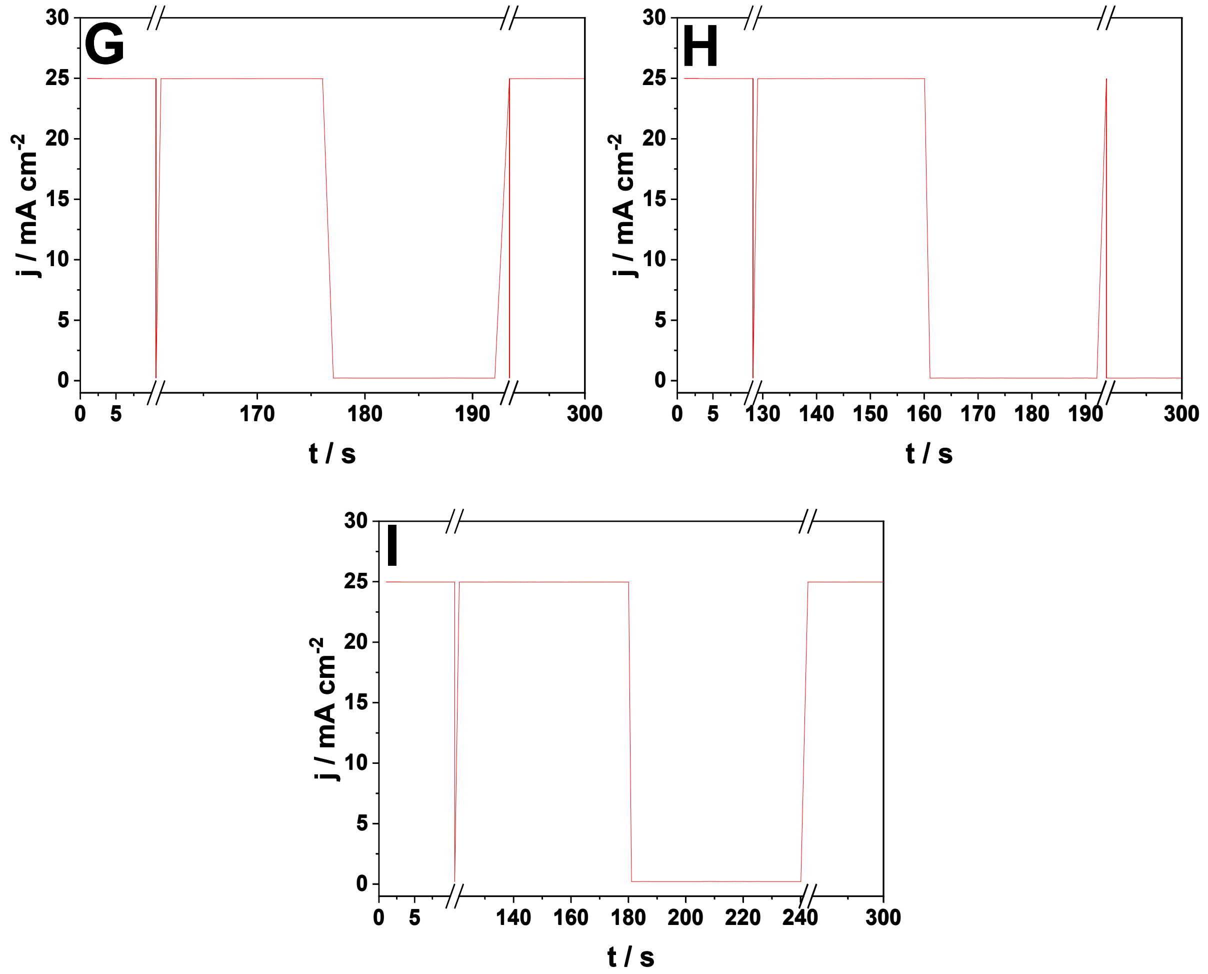

Figure S8. Applied current density profiles of H_2O_2 formation electrolysis applying constant current ($j_{constant}$= 25 mA cm^{-2} (A)) or pulsed current with different $t_{j_{high}}/t_{j_{low}}$ ratio: 0.5 s/0.5 s (B), 1 s/1 s (C), 2 s/2 s (D), 4 s/4 s (E), 8 s/8 s (F), 16 s/16 s (G), 32 s/32 s (H), 60 s/60 s (I). j_{high}= 25 mA cm^{-2}, j_{low}= 0.25 mA cm^{-2}; 5 min electrolysis.

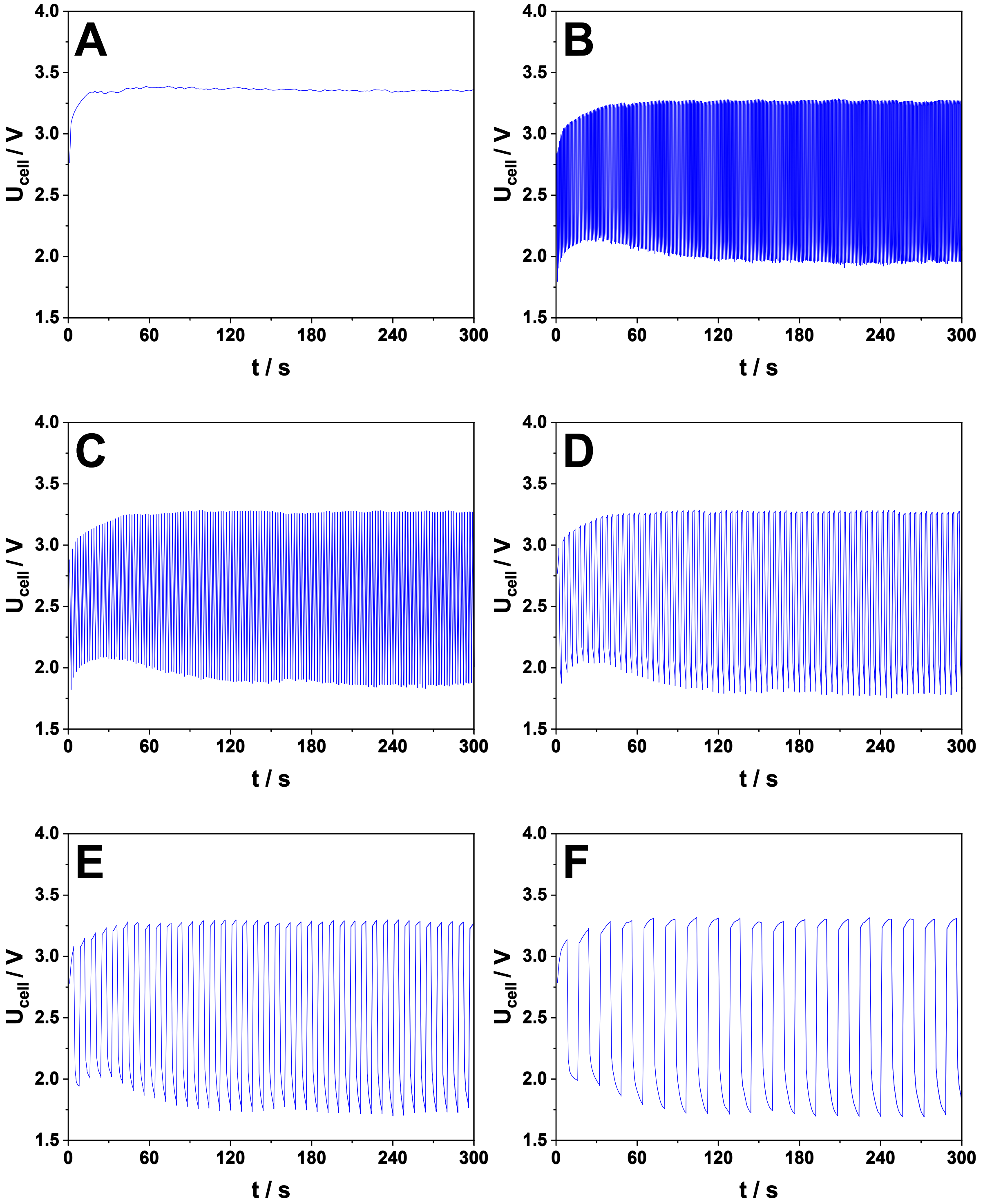
A
B
C
D
E
F
U_{cell} / V
t / s
4.0
3.5
3.0
2.5
2.0
1.5
0
60
120
180
240
300

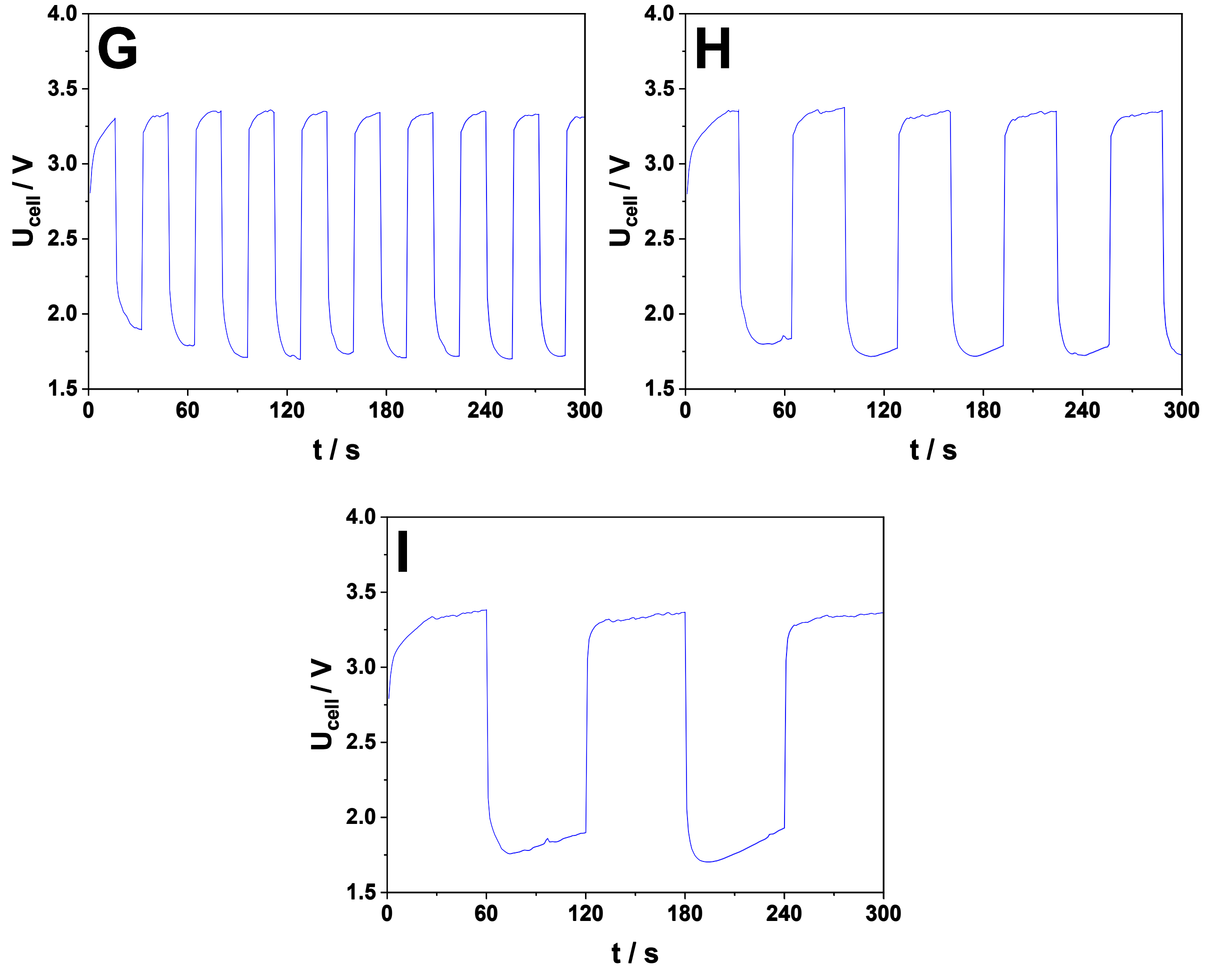

Figure S9. Measured cell voltages of H_2O_2 formation electrolysis applying constant current ($j_{constant}$= 25 mA cm^{-2} (A)) or pulsed current with different $t_{j_{high}}/t_{j_{low}}$ ratio: 0.5 s/0.5 s (B), 1 s/1 s (C), 2 s/2 s (D), 4 s/4 s (E), 8 s/8 s (F), 16 s/16 s (G), 32 s/32 s (H), 60 s/60 s (I). j_{high}= 25 mA cm^{-2}, j_{low}= 0.25 mA cm^{-2}; 2 M K_2CO_3; $V_{anolyte}$= 4.5 cm^3; single-pass anolyte, 0.75 cm^3 min^{-1}; 5 min electrolysis.

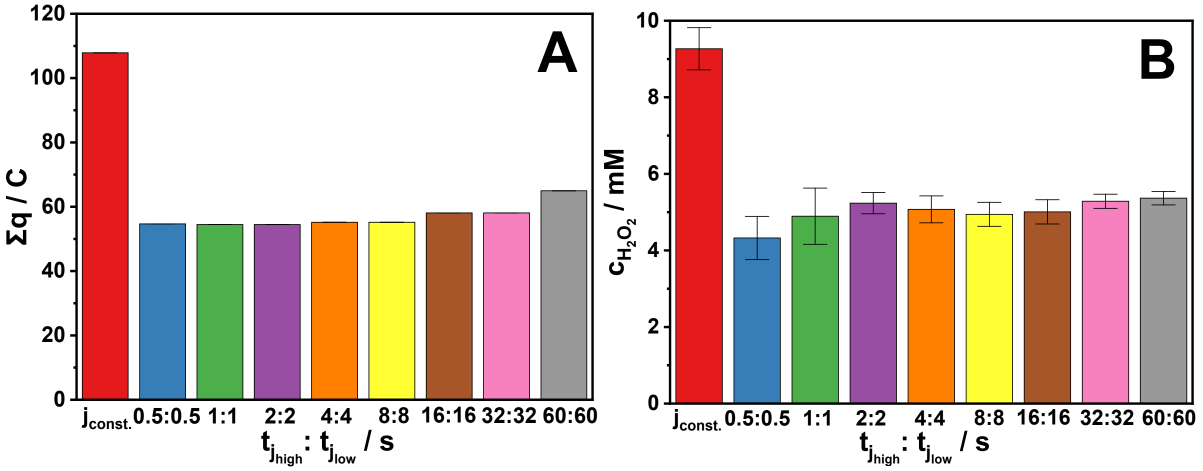

Figure S10. (A) Quantity of charge passed during H_2O_2 formation electrolysis and (B) concentration of produced H_2O_2 applying constant current ($j_{constant}$= 25 mA cm^{-2}) or pulsed current with different $t_{j_{high}}/t_{j_{low}}$ ratio: 0.5 s/0.5 s, 1 s/1 s, 2 s/2 s, 4 s/4 s, 8 s/8 s, 16 s/16 s, 32 s/32 s, 60 s/60 s. j_{high}= 25 mA cm^{-2}, j_{low}= 0.25 mA cm^{-2}; 2 M K_2CO_3; $V_{anolyte}$= 4.5 cm^3; single-pass anolyte, 0.75 cm^3 min^{-1}; 5 min electrolysis. The data of the column charts with error bars are based on the results of 3 electrolysis experiments.

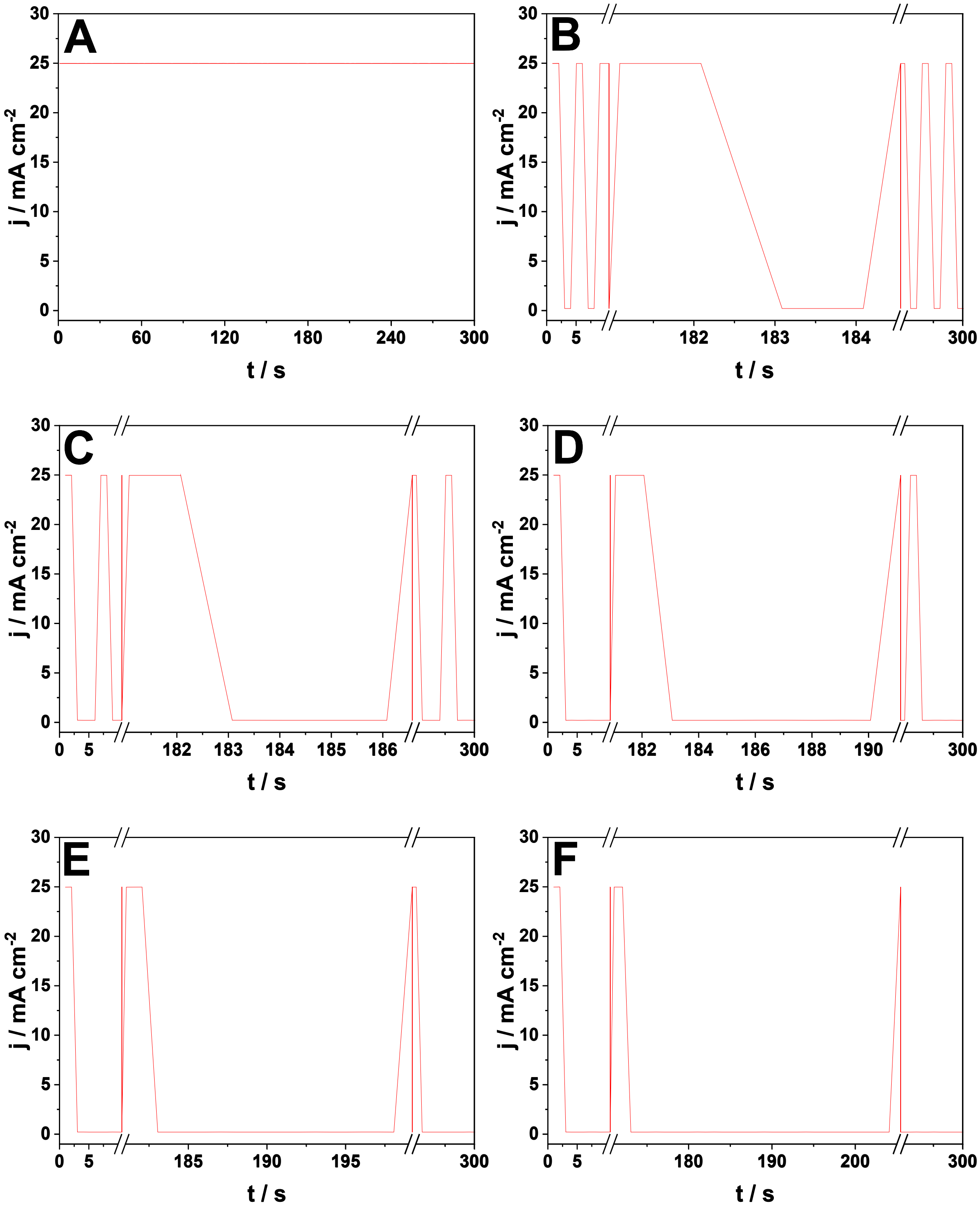

Figure S11. Applied current density profiles of H_2O_2 formation electrolysis applying constant current ($j_{constant}$= 25 mA cm^{-2}, (A)) or pulsed current with different $t_{j_{high}}/t_{j_{low}}$ ratio: $t_{j_{high}}$ = 2 s; $t_{j_{low}}$ = 2 s (B), 4 s (C), 8 s (D), 16 s (E) or 32 s (F). j_{high}= 25 mA cm^{-2}, j_{low}= 0.25 mA cm^{-2}; 5 min electrolysis.

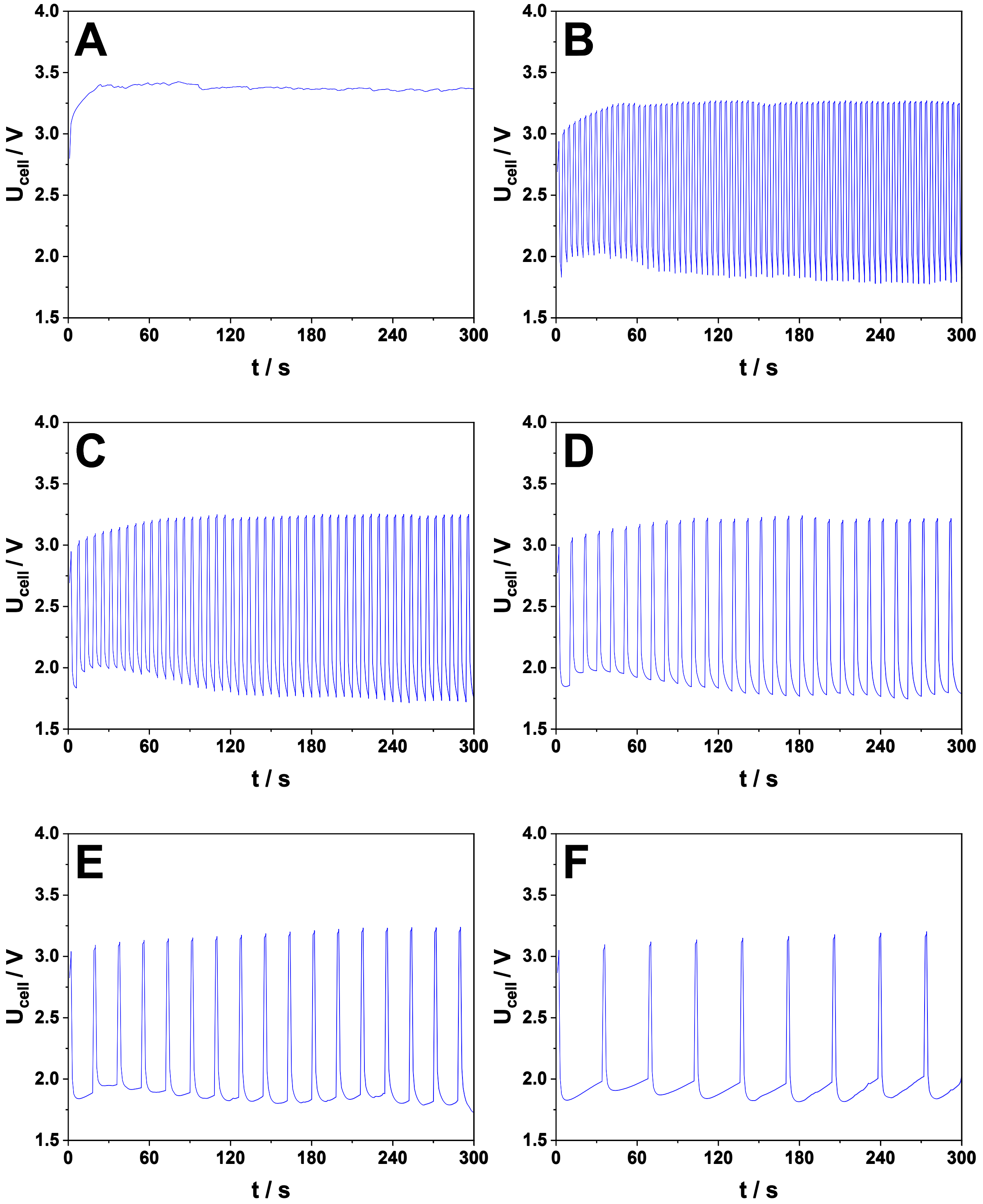

Figure S12. Measured cell voltages of H_2O_2 formation electrolysis applying constant current ($j_{constant}$= 25 mA cm^{-2}, (A)) or pulsed current with different $t_{j_{high}}/t_{j_{low}}$ ratio: $t_{j_{high}}$ = 2 s; $t_{j_{low}}$ = 2 s (B), 4 s (C), 8 s (D), 16 s (E) or 32 s (F). j_{high}= 25 mA cm^{-2}, j_{low}= 0.25 mA cm^{-2}; 2 M K_2CO_3; $V_{anolyte}$= 4.5 cm^3; single-pass anolyte, 0.75 cm^3 min^{-1}; 5 min electrolysis.

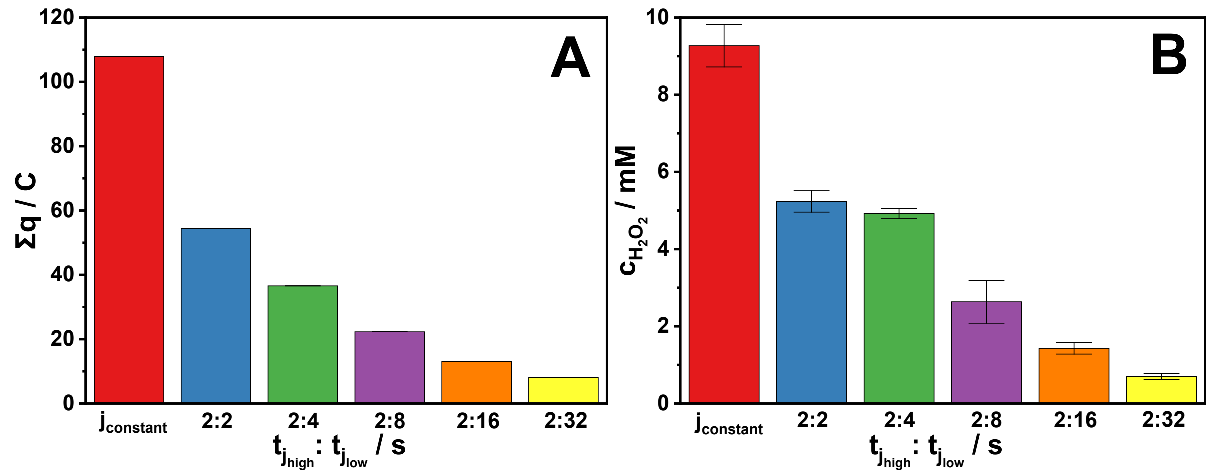

Figure S13. (A) Quantity of charge passed during H_2O_2 formation electrolysis and (B) concentration of produced H_2O_2 applying constant current ($j_{constant}$= 25 mA cm^{-2}) or pulsed current with different $t_{j_{high}}/t_{j_{low}}$ ratio: $t_{j_{high}}$ = 2 s; $t_{j_{low}}$ = 2 s, 4 s, 8 s, 16 s or 32 s. j_{high}= 25 mA cm^{-2}, j_{low}= 0.25 mA cm^{-2}; 2 M K_2CO_3; $V_{anolyte}$= 4.5 cm^3; single-pass anolyte, 0.75 cm^3 min^{-1}; 5 min electrolysis. The data of the column charts with error bars are based on the results of 3 electrolysis experiments.

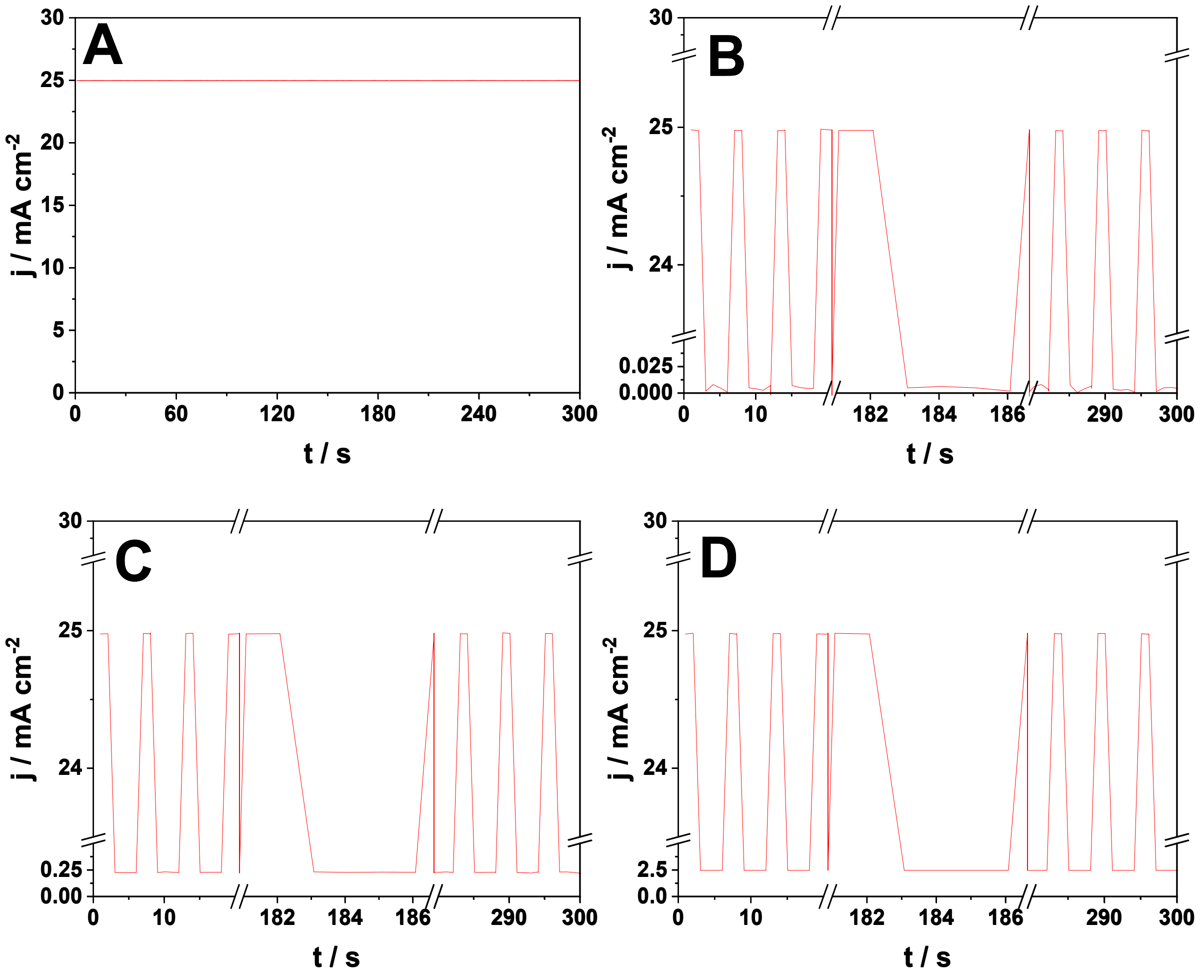

Figure S14. Applied current density profiles of H_2O_2 formation electrolysis applying constant current ($j_{constant}$= 25 mA cm^{-2} (A)) or pulsed current with different pulse amplitudes: $t_{j_{high}}/t_{j_{low}}$ = 2 s/4 s; j_{high}= 25 mA cm^{-2}, j_{low}= 0.025 (B), 0.25 (C) or 2.5 (D) mA cm^{-2}. 5 min electrolysis.

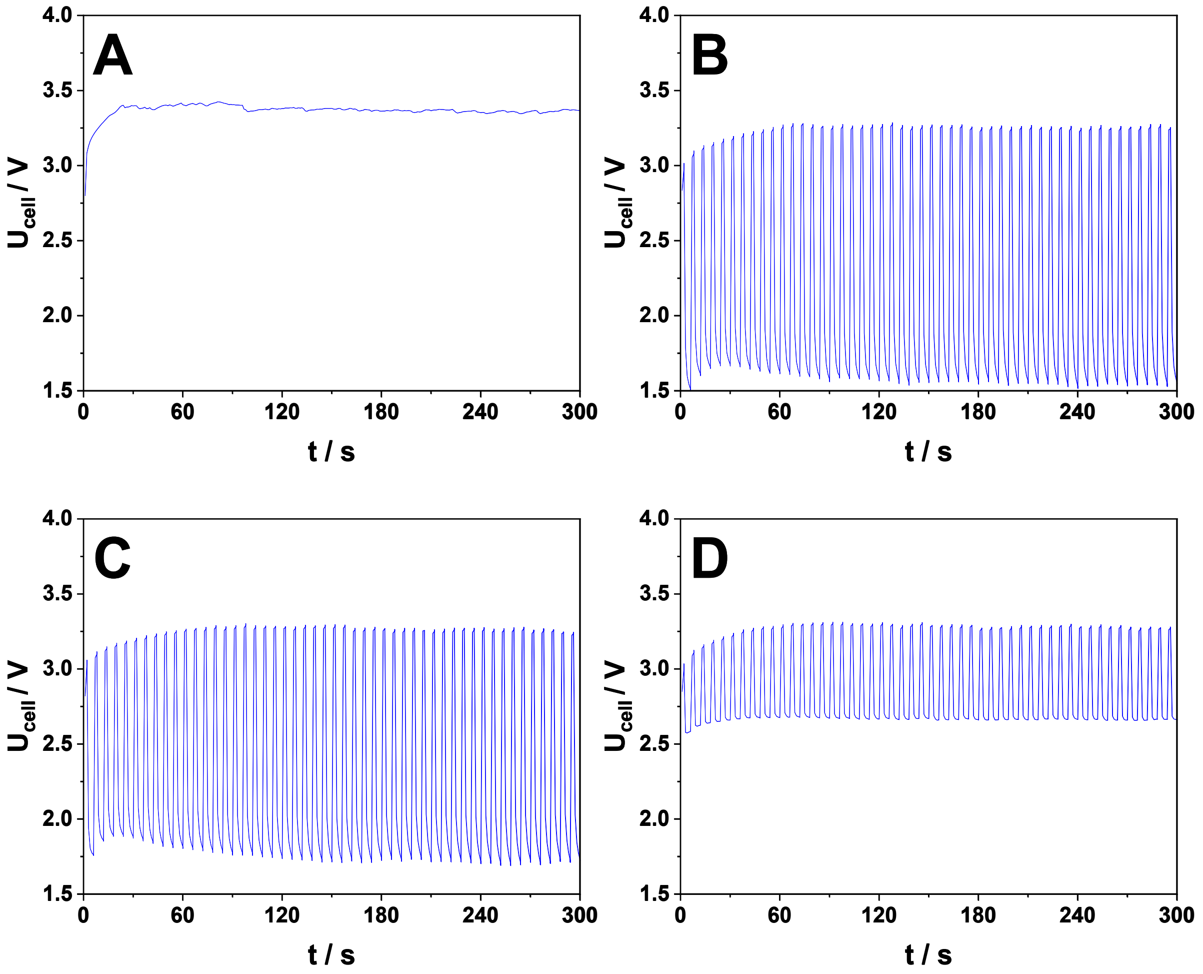

Figure S15. Measured cell voltages of H_2O_2 formation electrolysis applying constant current ($j_{constant}$= 25 mA cm^{-2} (A)) or pulsed current with different pulse amplitudes: $t_{j_{high}}/t_{j_{low}}$ = 2 s/4 s; j_{high}= 25 mA cm^{-2}, j_{low}= 0.025 (B), 0.25 (C) or 2.5 (D) mA cm^{-2}. 2 M K_2CO_3; $V_{anolyte}$= 4.5 cm^3; single-pass anolyte, 0.75 cm^3 min^{-1}; 5 min electrolysis.

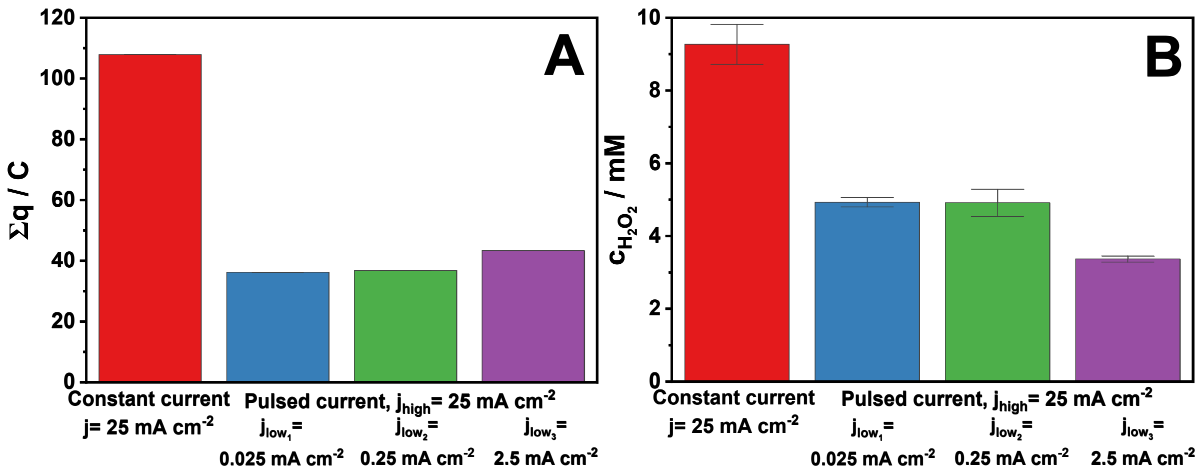

Figure S16. (A) Quantity of charge passed during H_2O_2 formation electrolysis and (B) concentration of produced H_2O_2 applying constant current ($j_{constant}$= 25 mA cm^{-2}) or pulsed current with different pulse amplitudes: $t_{j_{high}}/t_{j_{low}}$ = 2 s/4 s; j_{high}= 25 mA cm^{-2}, j_{low}= 0.025, 0.25 or 2.5 mA cm^{-2}. 2 M K_2CO_3; $V_{anolyte}$= 4.5 cm^3; single-pass anolyte, 0.75 cm^3 min^{-1}; 5 min electrolysis. The data of the column charts with error bars are based on the results of 3 electrolysis experiments.

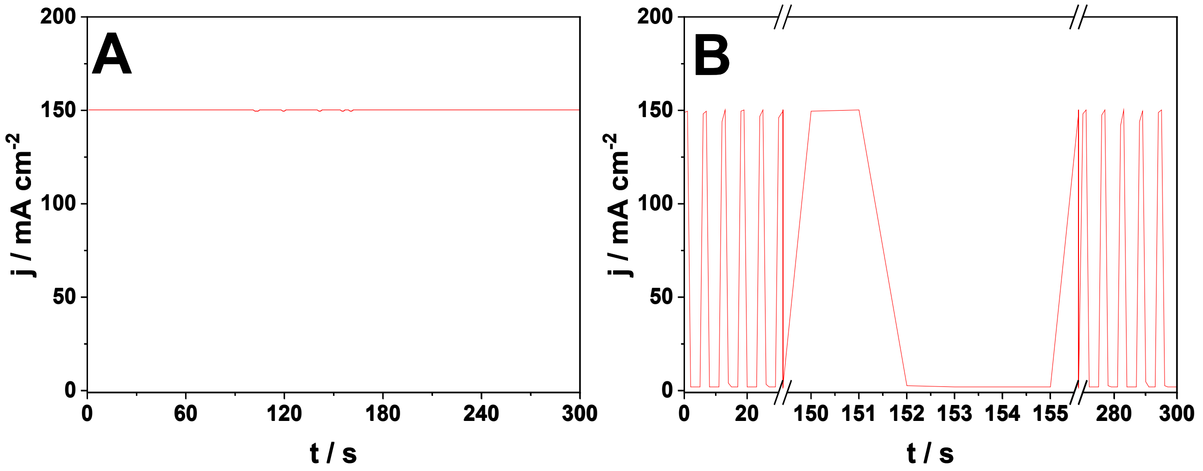

Figure S17. Applied current density profile of the H_2O_2 formation electrolysis applying (A) constant current ($j_{constant}$= 150 mA cm^{-2}) or (B) pulsed current ($t_{j_{high}}/t_{j_{low}}$ = 2 s/4 s; j_{high}= 150 mA cm^{-2}, j_{low}= 1.5 mA cm^{-2}). 5 min electrolysis.

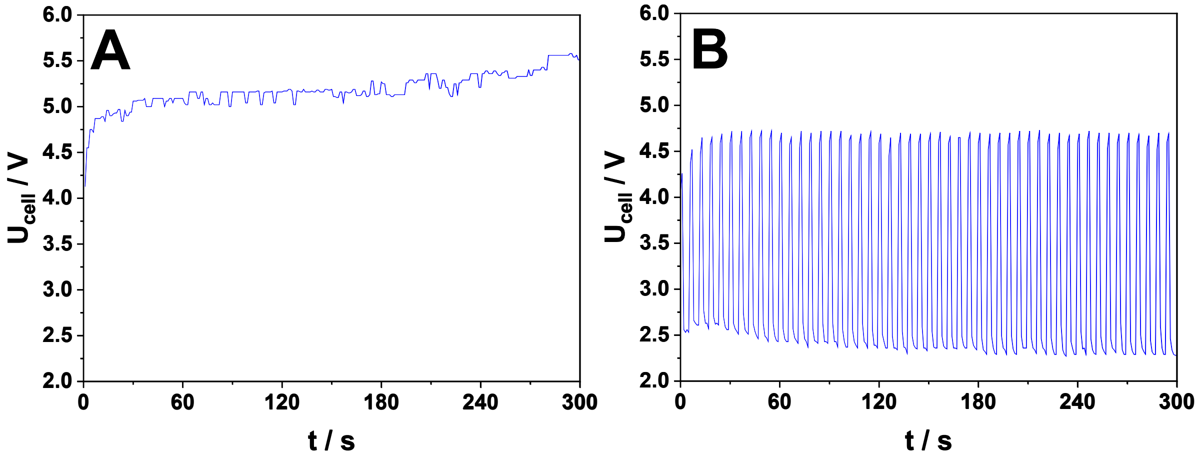

Figure S18. Measured voltages of the H_2O_2 formation electrolysis applying (A) constant current ($j_{constant}$= 150 mA cm^{-2}) or (B) pulsed current ($t_{j_{high}}/t_{j_{low}}$ = 2 s/4 s; j_{high}= 150 mA cm^{-2}, j_{low}= 1.5 mA cm^{-2}). 2 M K_2CO_3; $V_{anolyte}$= 4.5 cm^3; single-pass anolyte, 0.75 cm^3 min^{-1}; 5 min electrolysis.

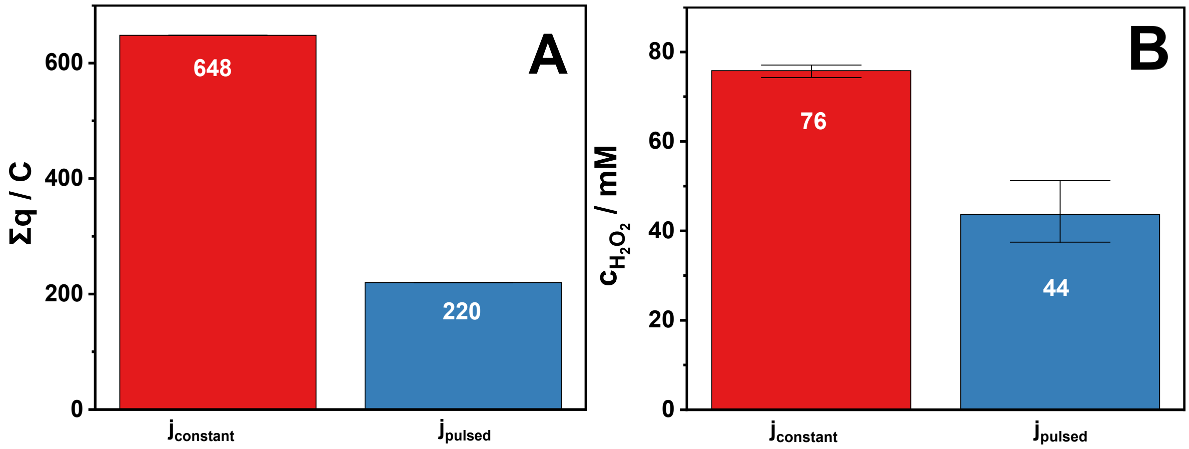

Figure S19. (A) Quantity of charge passed during H_2O_2 formation electrolysis and (B) concentration of produced H_2O_2 applying constant current ($j_{constant}$= 150 mA cm^{-2}) or pulsed current ($t_{j_{high}}/t_{j_{low}}$ = 2 s/4 s; j_{high}= 150 mA cm^{-2}, j_{low}= 1.5 mA cm^{-2}). 2 M K_2CO_3; $V_{anolyte}$= 4.5 cm^3; single-pass anolyte, 0.75 cm^3 min^{-1}; 5 min electrolysis. The data of the column charts with error bars are based on the results of 3 electrolysis experiments.

Quantification of H_2O_2 electrochemically oxidized to O_2

(from data in Fig. 6 and Fig. S13. A and B)

Experimental data

Constant current electrolysis
$\bar{x}FE_{j_{constant}} = 7.5\ \%$
$q_{j_{constant}} = 107.9\ C$
$\bar{x}n_{H_2O_2}(\text{measured}) = 4.2 \times 10^{-5}\ \text{mol}$

Pulsed current electrolysis
$\bar{x}FE_{j_{pulsed}} = 11.7\ \%$
$q_{j_{pulsed}} = 36.6\ C$
$\bar{x}n_{H_2O_2} = 2.2 \times 10^{-5}$

Calculation

FE calculation
$FE = \frac{n_{H_2O_2} \times z \times F}{q} \times 100$
$z = 2$
$F = 96485\ C\ mol^{-1}$

Calculation of $n_{H_2O_2}$ if $\bar{x}FE_{j_{constant}} = \bar{x}FE_{j_{pulsed}}$
$n_{H_2O_2}(\text{theoretical}) = 6.5 \times 10^{-5}\ \text{mol}$

Based on the calculation above, about 1/3 of the amount of H_2O_2 produced is being electrochemically oxidized, if constant current electrolysis is applied instead of pulsed current electrolysis.

Calculation of energy efficiency (EE)

Constant current electrolysis

$\bar{x}n_{H_2O_2}$= 3.41 × 10^{-4} mol
$\bar{x}p$= 0.0108 kW
t= 0.083 h
W= 8.964 × 10^{-4} kWh
EE= 0.38 $mol_{H_2O_2}$ kWh^{-1}

Pulsed current electrolysis

$\bar{x}n_{H_2O_2}$= 1.96 × 10^{-4} mol
$\bar{x}p$= 0.0038 kW
t= 0.083 h
W= 3.154 × 10^{-4} kWh
EE= 0.62 $mol_{H_2O_2}$ kWh^{-1}

The increase of EE is ca. 63 % for the pulsed current electrolysis compared to constant current electrolysis (EE is 1.63 times higher for electrolysis with j_{pulsed} vs. $j_{constant}$).

Based on ref.[1], the energy efficiency of the H_2O_2 production process on industrial level ranges between 1.7-2.3 $mol_{H_2O_2}$ kWh^{-1}. This means that the energy consumption is between 12.8-17.6 kWh $kg^{-1}_{H_2O_2}$.

According to ref.[2], the specific energy consumption of H_2O_2 production increases from ca. 19 kWh $kg^{-1}_{H_2O_2}$ up to 40 kWh $kg^{-1}_{H_2O_2}$ by increasing the applied current density from 100 mA cm^{-2} up to 700 mA cm^{-2} within an $FE_{H_2O_2}$ range of 50-35 % using a BDD plate anode electrode in a non-zero gap flow cell with a 2 M K_2CO_3 aqueous anolyte.

<u>Energy consumption calculation</u>

$M_{H_2O_2}$= 34 g mol^{-1}

Constant current electrolysis

77.4 kWh $kg^{-1}_{H_2O_2}$

Pulsed current electrolysis

47.4 kWh $kg^{-1}_{H_2O_2}$

In our study, the energy consumption at 150 mA cm^{-2} with constant current operation is 77.4 kWh $kg^{-1}_{H_2O_2}$ while it is 47.4 kWh $kg^{-1}_{H_2O_2}$ with pulsed current. Although the latter is 2.5 times higher than the most optimized value described in the reference cited above, it is important to note that the highest $FE_{H_2O_2}$ in the present study was only 17 % (compared to 50 %) due to the non-optimized electrode properties (boron doping of 1600 ppm only) and operational parameters (electrode and cell design). By optimizing electrode and cell properties and process parameters, it is expected that the energy consumption will be decreased, ideally to an economically feasible level.

References

[1] G. Goor, J. Glenneberg, S. Jacobi, J. Dadabhoy, E. Candido, Hydrogen Peroxide, *Ullmann's Encyclopedia of Industrial Chemistry*, pp. 1-40.

[2] D. Pangotra, L. I. Csepei, A. Roth, V. Sieber, L. Vieira, *Green Chem.* **2022**, *24*, 7931–7940.
